# Guest-dependent single-ion magnet behaviour in a cobalt(ii) metal–organic framework†
†Electronic supplementary information (ESI) available: Preparation and physical characterization data. Crystallographic refinement details. Additional figures (Fig. S1–S23) and tables (Tables S1–S4). CCDC 1415915–1415921. For ESI and crystallographic data in CIF or other electronic format see DOI: 10.1039/c5sc04461h


**DOI:** 10.1039/c5sc04461h

**Published:** 2015-12-10

**Authors:** Julia Vallejo, Francisco R. Fortea-Pérez, Emilio Pardo, Samia Benmansour, Isabel Castro, J. Krzystek, Donatella Armentano, Joan Cano

**Affiliations:** a Departament de Química Inorgànica , Instituto de Ciencia Molecular (ICMOL) , Universitat de València , 46980 Paterna , València , Spain . Email: emilio.pardo@uv.es ; Email: joan.cano@uv.es; b National High Magnetic Field Laboratory , Florida State University , Tallahassee , Florida 32310 , USA; c Dipartimento di Chimica e Tecnologie Chimiche , Università della Calabria , 87036 , Arcavacata di Rende , Cosenza , Italy; d Fundació General de la Universitat de València (FGUV) , Spain

## Abstract

Single-ion magnets (SIMs) are the smallest possible magnetic devices for potential applications in quantum computing and high-density information storage. Both, their addressing in surfaces and their organization in metal–organic frameworks (MOFs) are thus current challenges in molecular chemistry. Here we report a two-dimensional 2D MOF with a square grid topology built from cobalt(ii) SIMs as nodes and long rod-like aromatic bipyridine ligands as linkers, and exhibiting large square channels capable to host a large number of different guest molecules. The organization of the cobalt(ii) nodes in the square layers improves the magnetic properties by minimizing the intermolecular interactions between the cobalt(ii) centres. Moreover, the SIM behaviour was found to be dependent on the nature of the aromatic guest molecules. The whole process could be followed by single-crystal X-ray diffraction, providing comprehensive evidence of the putative role of the solvent guest molecules that leave a “fingerprint” on the 2D structures and thus, on the cobalt environment.

## Abstract 2

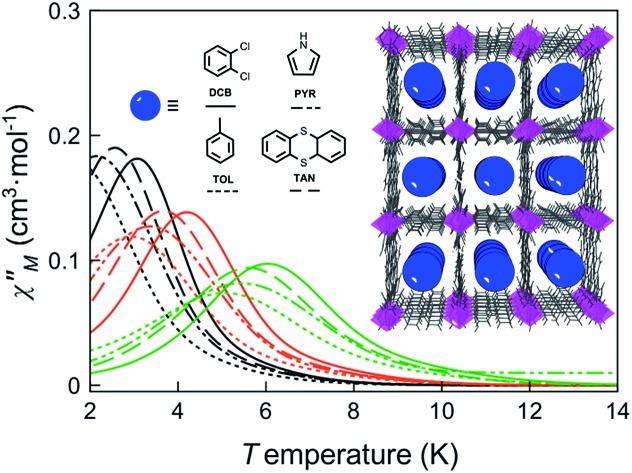
Such exciting properties as porosity and single-ion magnetic behavior are merged into a single unique material which affords the first in-depth study of guest-dependent SIM behavior in a MOF.

## Introduction

Single-molecule magnets (SMMs),^1^,^2^ and more recently, also the related single-ion magnets (SIMs) – both showing potential applications as high-density magnetic memory and quantum computing devices in the emerging field of molecular spintronics^3^,^4^ – attract the attention of many groups working in the fields of Molecular Magnetism^5^ and Multifunctional Materials.^6^–^8^ Among them, SIMs have gained an especial relevance in recent years, and different examples with lanthanides^9^–^13^ and actinides,^14^–^17^ but also with first^18^–^25^ and third^26^ row transition metal ions have been reported.

Because of their fascinating structures and, especially, the wide range of physical (optical, magnetic, electronic, *etc.*) and chemical properties (gas storage and separation, transport, catalysis, *etc.*) that they can exhibit, extended multidimensional porous coordination polymers, also known as metal-organic frameworks (MOFs),^27^–^32^ have also attracted the interest of many groups for the design of multifunctional materials.^6^ For example, the rich host–guest chemistry of spin crossover metal–organic frameworks (SCO-MOFs),^33^–^35^ which is related to the intrinsic porous character of MOFs,^36^,^37^ allows these systems to have excellent potential application in molecular magnetic recognition.^38^

**Scheme 1:**
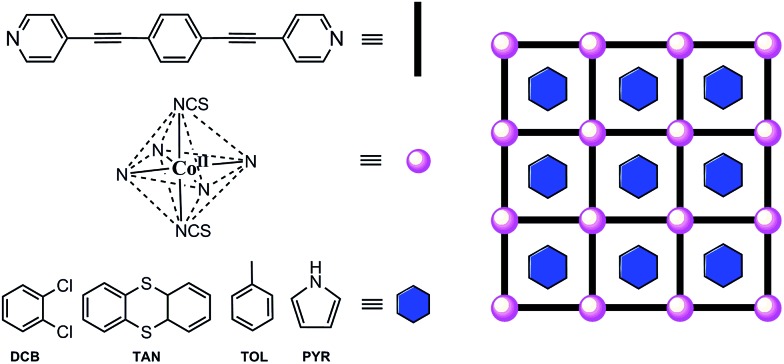
Ligand design approach to cobalt(ii)-based SIM-MOFs for magnetic sensing of aromatic compounds.

Aiming at sticking together these two hot subjects in chemistry in the same material and also by the possible resulting properties, we designed the synthesis of a MOF built from the repetition of highly organized SIMs, referred to as SIM-MOF.^39^ Moreover, we have taken advantage of the intrinsic porosity of the compound to tune the slow magnetic relaxation effects of the SIM nodes. So, we chose a rod-like aromatic bipyridine ligand with a very long organic spacer, such as 1,4-bis(pyridine-4-ylethynyl)benzene^40^ (bpeb), capable of acting as a bis(monodentate)bridging ligand toward magnetically anisotropic high-spin cobalt(ii) ions to: (i) yield square grid-type two-dimensional (2D) MOFs with a porous structure possessing relatively large channels occupied with potentially exchangeable aromatic guest molecules that would preclude for undesired layer interpenetration, and (ii) afford the appropriate ligand field for observing SIM behaviour while preventing any magnetic interaction among the SIM metal nodes^41^–^43^ (Scheme 1).

In this work, we report the synthesis, structural, high-frequency and high-field EPR (HFEPR) spectroscopic and magnetic characterisation, as well as theoretical calculations, of a novel series of two-dimensional (2D) MOFs of general formula [Co(bpeb)_2_(NCS)_2_]·*n*G, where G stands for the *ortho*-dichlorobenzene (**DCB@1** with *n* = 7), thianthrene (**TAN@1** with *n* = 4), toluene (**TOL@1** with *n* = 6), and pyrrole (**PYR@1** with *n* = 8) guest molecules. Compounds **G@1** (G = DCB, TAN, TOL, and PYR) can be described as an extended array of nanostructured octahedral cobalt(ii) SIMs, thus constituting one of the very first examples of SIM-MOFs.^39^,^41^–^44^ Interestingly, as a direct consequence of the replacement of the DCB guest molecules hosted in the channels of the original compound (**DCB@1**) along this unique series of adsorbates, tuning of the SIM behaviour takes place depending on the nature of the guest.

## Results and discussion

### Synthesis and X-ray crystal structure

Compound **DCB@1** was first obtained as well-formed rectangular prisms by layering a methanol solution of Co^II^(NCS)_2_ over an *ortho*-dichlorobenzene/methanol solution (4 : 1 v/v) of bpeb ligand (1 : 2 metal–ligand molar ratio) in an essay tube at room temperature (see Experimental section). Thereafter, **TAN@1**, **TOL@1**, and **PYR@1** were obtained through a single-crystal-to-single-crystal (SC-SC) transformation by immersing crystals of **DCB@1** for a week in the corresponding solvents thianthrene, toluene, and pyrrole, respectively (Table S1, ESI†).

**Fig. 1:**
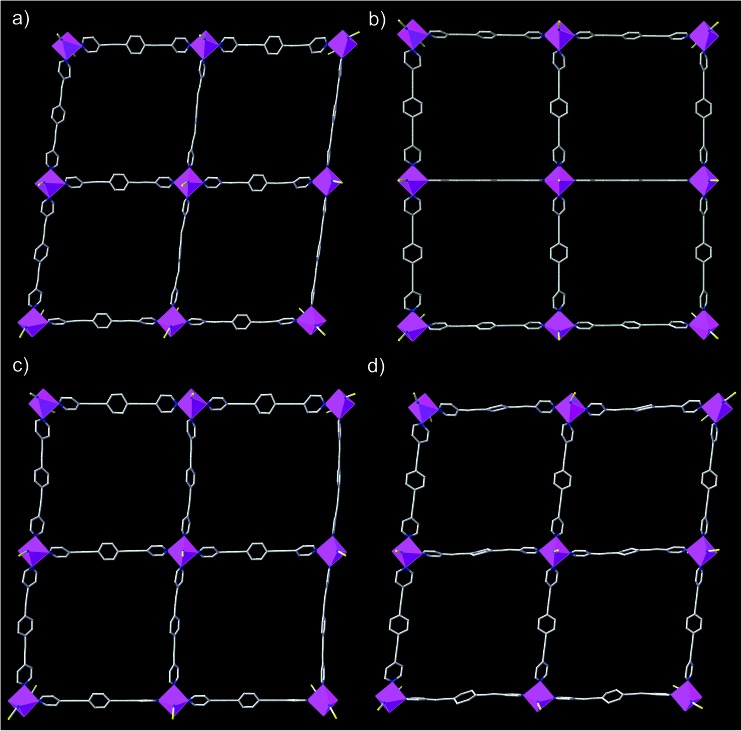
Perspective view of a fragment of the neutral square grid-type flat layers of **DCB@1** (a), **TAN@1** (b), **TOL@1** (c), and **PYR@1** (d). Cobalt atoms from the coordination network are represented by purple polyhedra whereas the ligands are depicted as sticks (hydrogen atoms are omitted for clarity).

The structures of **DCB@1**, **TAN@1**, **TOL@1**, and **PYR@1** were determined by X-ray diffraction on single crystals at 100 K (see Experimental section), showing that they are different polymorphs. All four compounds are made up of similar square grid flat layers of (4^4^·6^2^) net topology^43^–^46^ and free highly disordered solvent molecules (Fig. 1 and S1–S3, ESI†). However, the different nature of the guests hosted in the channels together with the intrinsic flexibility of the system, promote more or less severe distortions from the ideal square grid structure avoiding isostructurality (Fig. 1 and S3†). The estimated volumes of accessible solvent voids (Fig. S4†) are *ca.* 65% of the total unit cell volume for all four compounds (see ESI for details†).

**Fig. 2:**
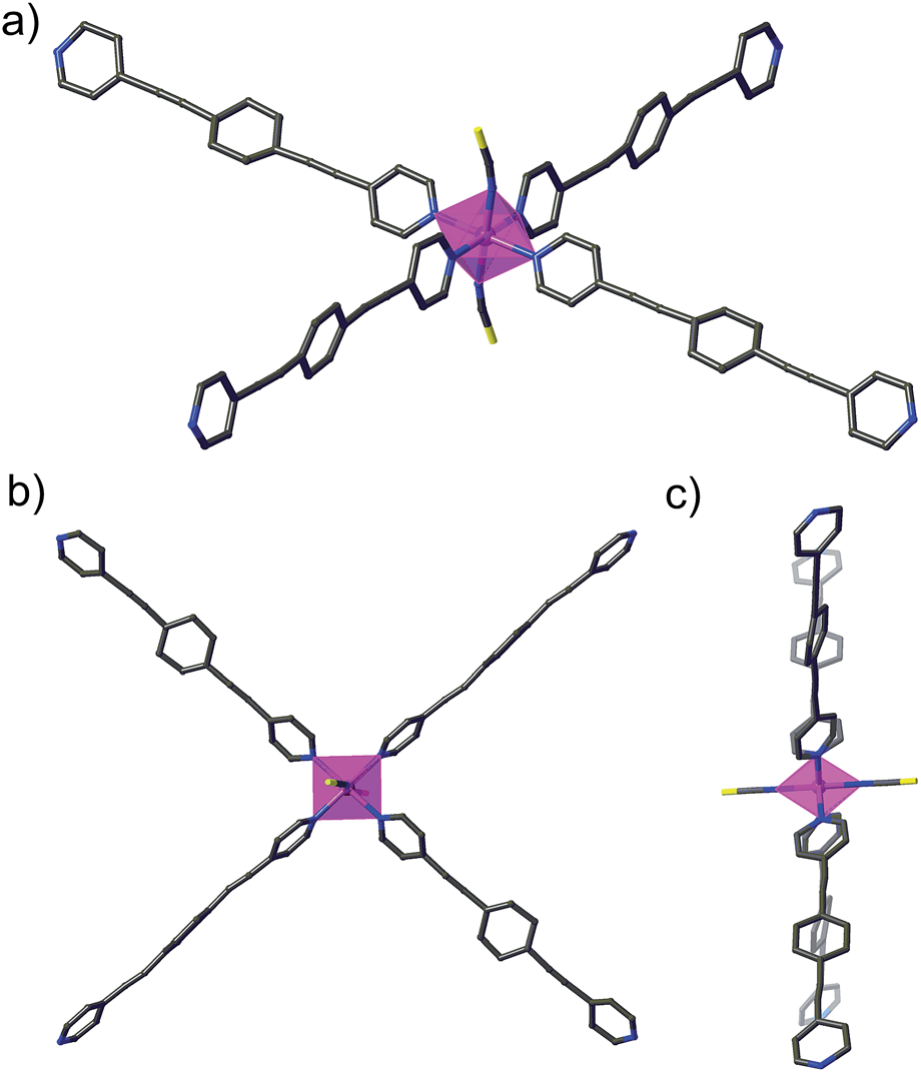
Perspective (a), cenital (b) and side (c) views of the cobalt(ii) environment in **DCB@1**.

The cobalt(ii) ions of **DCB@1**, **TAN@1**, **TOL@1**, and **PYR@1** are located in the corners of each square grid and linked by the very long organic spacers acting as edges of the squares, as illustrated in Fig. 1, 2 and S3† for the parent compound of the series. In fact, the length of the rod-like bpeb bridging ligand in **DCB@1**, **TAN@1**, **TOL@1**, and **PYR@1** results in the Co^II^ ions in each square grid being very well isolated (see Table S2†). In contrast, neighbouring Co^II^ ions belonging to adjacent layers are closer to each other [shortest intermetallic distances are in the range of 11.502(2)–11.760(2) Å] (see Table S2†) but still quite well isolated.

In all four compounds, each cobalt(ii) ion is situated in a highly distorted six-coordinated octahedral environment, CoN_6_. The equatorial Co–N bond distances are somewhat larger than the axial Co–N bond distances (see Table S2†), all of them being typical of high-spin Co^II^ ions. This leads to an overall tetragonal distortion (axial compression) of the octahedral metal environment (Fig. S5 and Table S2†). Interestingly, the nature of the guest induces severe distortions in the organic spacer conformation, which is also apparent in the structural dimensions of the square layers of **DCB@1**, **TAN@1**, **TOL@1**, and **PYR@1** (Fig. 1 and S3†). The main structural variations along this series are related to the values of the dihedral angle between the terminal pyridine and central benzene rings (*δ*), resulting in distortions also in the values of the intralayer cobalt(ii)–cobalt(ii)–cobalt(ii) angle (*φ*) (see Table S2†).

Ultimately, and as a consequence of these distortions, the environment of the cobalt(ii) ions changes significantly from one compound to another, affording further proof of the flexibility of these systems. It can be envisaged that the reported guest-dependent structural changes may have a great influence on those physical properties strongly dependent of the metal coordination environment such as the dynamic magnetic properties of the cobalt(ii) centres (see discussion below). In this regard, it should be highlighted that the reported crystal structures are collected at 100 K whereas dynamic magnetic properties are usually measured below 10 K. However, although small structural changes may take place between 10 and 100 K, the crystal structures show unambiguously that the nature of the guest molecule strongly modifies the cobalt(ii) environment at 100 K, which must also apply below 10 K, even if temperature dependent structural changes may also occur.

### Physical characterisation

The solvent content of **DCB@1**, **TAN@1**, **TOL@1**, and **PYR@1** was determined by elemental (C, H, N, and S) analysis (see Experimental section) and further confirmed by thermogravimetric analysis (TGA) under a dry N_2_ atmosphere (Fig. S6†). The mass loss values are consistent with 7 DCB (**DCB@1**), 4 TAN plus 4 MeOH (**TAN@1**), 6 TOL (**TOL@1**), and 8 PYR (**PYR@1**) molecules per formula unit, which are in agreement with the formulas determined by elemental analysis (ESI†).

**Fig. 3:**
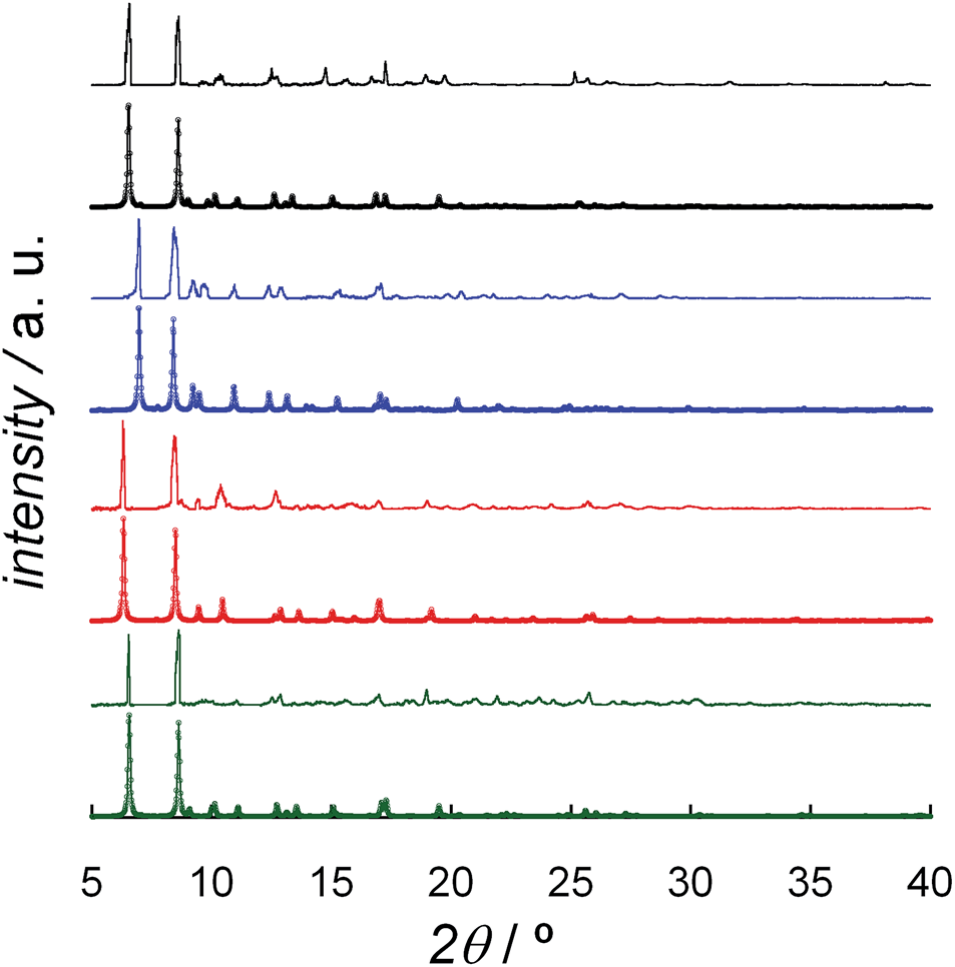
Experimental (top) and calculated (bottom) XRPD pattern profiles of **DCB@1** (green), **TAN@1** (red), **TOL@1** (blue) and **PYR@1** (black) in the 2*θ* range of 5.0–40.0° at 100 K.

The powder X-ray diffraction (PXRD) patterns of freshly prepared samples of **DCB@1**, **TAN@1**, **TOL@1**, and **PYR@1** (measured as suspensions of the samples in the corresponding solvent solutions) are similar but not identical, and they are all consistent with the calculated ones (Fig. 3). These features confirm both that the bulk samples are isostructural to the crystals selected for single-crystal X-ray diffraction, and that the three adsorbates (**TAN@1**, **TOL@1** and **PYR@1**) are different polymorphs of the original compound (**DCB@1**). The different degree of distortion in the organic spacer conformation – arising from the different nature of the guest molecule in each compound – lies at the origin of the lack of isostructurality.

Finally, specific heat capacity measurements were carried out for **DCB@1**, **TAN@1**, **TOL@1**, and **PYR@1** in the temperature range of 100.0–2.0 K. The lack of *λ* peaks for all compounds (Fig. S7†) suggests that no phase transition occurs below 100 K.

### Static (dc) magnetic properties and HFEPR measurements

The magnetic properties of **DCB@1**, **TAN@1**, **TOL@1** and **PYR@1** were also measured using suspensions of the samples in the corresponding solvent solutions to prevent any possible desolvation that could modify the magnetic properties.

The direct current (dc) magnetic properties of frozen matrix solvent solutions of **DCB@1**, **TAN@1**, **TOL@1** and **PYR@1** in the form of the *χ*_M_*T versus T* plots (*χ*_M_ being the dc magnetic susceptibility per Co^II^ unit) showed qualitatively similar behaviour (Fig. S8†). At 100 K, the *χ*_M_*T* values vary in the range of 2.42–2.58 cm^3^ mol^–1^ K, being within the range expected for a high-spin d^7^ Co^II^ (*S* = 3/2) ion with some orbital momentum contribution. Upon cooling, *χ*_M_*T* continuously decreases to reach values in the range of 1.87–1.90 cm^3^ mol^–1^ K at 2.0 K (Fig. S8†). This magnetic behaviour reveals not only the occurrence of significant spin–orbit coupling (SOC) in all four compounds but also the expected lack of magnetic coupling transmission both through the selected long organic spacer and between adjacent layers, meaning the Co centres are perfectly isolated.

The magnetic susceptibility data of octahedral cobalt(ii) complexes should be analysed on the SOC framework. At sufficiently low temperatures (below *ca.* 100 K), when only the two most stable *J* states are solely (or almost solely) populated, an approach considering an *S* = 3/2 showing large *g*-factors and zero-field splitting (ZFS) parameters arising from a significant SOC can be used.^47^ In such a case, significant temperature independent paramagnetism (TIP) must be considered in order to simulate the depopulation of the higher *J* excited states coming from the SOC. Depending on the order of the *J* states, this approach can be occasionally used at higher temperatures.

The magnetic susceptibility data below 100 K of **DCB@1**, **TAN@1**, **TOL@1** and **PYR@1** were then analysed by using the appropriate spin Hamiltonian for an isolated *S* = 3/2 with large ZFS,

1 *H* = *D*[*S*_z_^2^ + (1/3)*S*(*S* + 1)] + *βHgS*,

which takes into account the axial magnetic anisotropy (*D*) of the tetragonally distorted high-spin d^7^ Co^II^ ion. The values obtained from the least-squares fit of the magnetic susceptibility through the VPMAG program^48^ are collected in Table 1. The theoretical curves match the experimental data in the whole temperature range (solid lines in Fig. S8†). The large |*D*| values observed for **DCB@1**, **TAN@1**, **TOL@1** and **PYR@1** (Table 1) are similar to those found in other reported cobalt(ii) SIMs. Moreover, they agree fairly well with those found from NEVPT2 calculations on the experimental geometries (see Table 1 and computational details in ESI†), which allow the assigning of a positive sign to the *D* values.^22^,^49^ The discrepancies among the individual experimental and theoretical *D* values arise most likely as a consequence of both the certain inaccuracy of the theoretical method and the subtle modifications of the cobalt(ii) coordination environments at low temperature with respect to the crystal structures used in the theoretical calculations. However, they show the same overall trend as follows: **DCB@1** < **TAN@1** < **PYR@1** < **TOL@1** (Table 1). This is illustrated in Fig. S9† which shows an almost perfect linear correlation between both experimental and theoretical *D* values.

**Table 1:** Selected experimental^*a*^ and theoretical magnetic data for **DCB@1**, **TAN@1**, **TOL@1**, and **PYR@1**

Compound	*g* ^*b*^	|*D*|^*c*^ (cm^–1^)	TIP × 10^6^	*F* ^*d*^	*D* ^*e*^ (cm^–1^)
**DCB@1**	2.563(1)	64.9(9)	–2310(50)	2.0 × 10^–5^	91.2
**TAN@1**	2.590(1)	67.1(9)	–4550(90)	1.7 × 10^–5^	95.7
**TOL@1**	2.583(1)	84.4(4)	–1200(8)	3.5 × 10^–6^	117.9
**PYR@1**	2.595(1)	70.3(9)	–1010(30)	1.4 × 10^–5^	99.8

^*a*^Standard deviations are given in parentheses.

^*b*^Landé factor (see eqn (1)).

^*c*^Axial magnetic anisotropy obtained from the fit of the magnetic susceptibility data (see eqn (1)).

^*d*^Agreement factor defined as ∑[(*P*)_exp_ – (*P*)_calcd_]^2^/∑[(*P*)_exp_]^2^, where *P* is the physical property under study.

^*e*^Axial magnetic anisotropy obtained from NEVPT2 calculations.

**Fig. 4:**
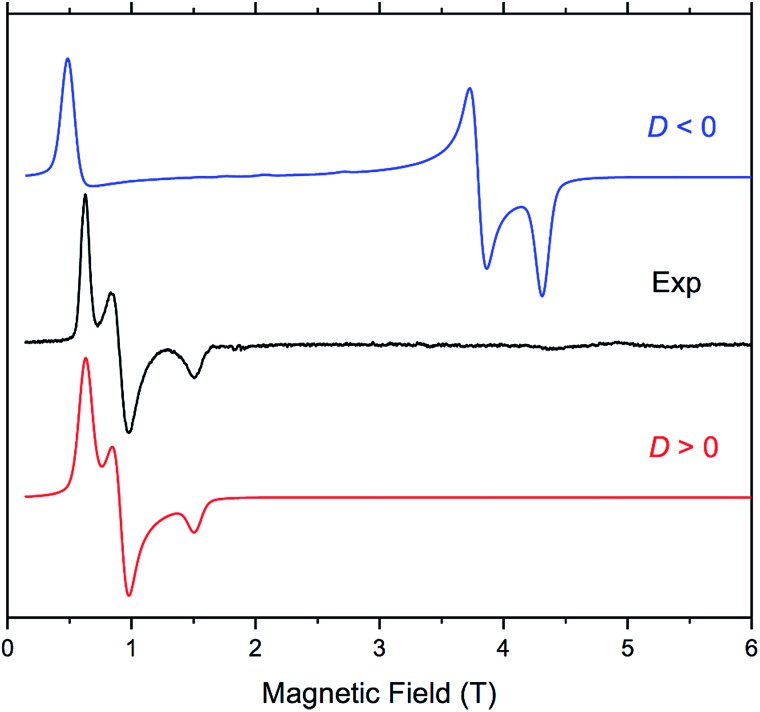
EPR spectrum of **TAN@1** at 53.2 GHz and 4.5 K (black trace) accompanied by powder-pattern simulations (colored traces) using the following spin Hamiltonian parameters: *S* = 3/2; |*E*/*D*| = 0.125, and *g* = [2.57, 2.57, 2.63]. Blue trace: *D* < 0; red trace: *D* > 0. The rhombicity factor represents the upper bound since it is correlated with the *g*_*x*_ and *g*_*y*_ value.

The four complexes were also investigated by HFEPR. Given the large magnitude of *D* as suggested by susceptibility measurements and theoretical results, HFEPR was not expected to deliver its value; rather, we hoped to determine its sign, and the rhombicity factor (*E*/*D*) of the ZFS tensor. Indeed, two samples, **DCB@1** and **TAN@1**, both kept in (frozen) solution to avoid loss of the guest molecules, produced fine powder-patterned HFEPR spectra at low temperatures (Fig. 4 and S10–S12†). Rather than using the effective *S* = 1/2 spin Hamiltonian in interpreting these spectra, we used the *S* = 3/2 spin Hamiltonian, assuming an arbitrarily large value of *D* compared to the sub-THz wave frequency. HFEPR, in agreement with the NEVPT2 calculations, established a positive sign of *D* and delivered the values of rhombicity factor |*E*/*D*| plus the components *g*_i_ of the *intrinsic S* = 3/2 *g*-tensor, shown in Table 2.

**Table 2:** Selected experimental spectroscopic and magnetic data for **DCB@1**, **TAN@1**, **TOL@1**, and **PYR@1**

Compound	*g*_*x*_ ^*a*^	*g*_*y*_ ^*a*^	*g*_*z*_ ^*a*^	*g*_*x*_ ^*b*^	*g*_*y*_ ^*b*^	*g*_*z*_ ^*b*^	*E*/*D*^*b*^
**DCB@1**	4.05	6.05	2.45	2.54	2.56	2.59	0.130
**TAN@1**	4.06	6.00	2.48	2.52	2.56	2.59	0.125
**TOL@1**	3.90	5.90	2.50	—	—	—	∼0.110
**PYR@1**	4.09	6.10	2.52	—	—	—	∼0.075

^*a*^ *g*_i_ values of the ground Kramer's doublet extracted from the fit of the magnetisation curves (**DCB@1** and **TAN@1**) or the HFEPR spectra (**TOL@1** and **PYR@1**).

^*b*^ *g*_i_ and *E*/*D* values of the anisotropic *S* = 3/2 ground state from the simulation of the HFEPR spectra.

HFEPR measurements of samples of **PYR@1** and **TOL@1** were not as successful, however, since the pronounced crystallinity of these samples prohibited obtaining ideal powder spectra. Still, positive values of *D* were confirmed in these two cases, and the rhombicity factor evaluated (Fig. S13 and S14†). The usual method of dealing with crystalline samples, *i.e.* grinding, was problematic due to the presence of solvent, and brought no improvement.

The dc magnetisation data in the form of the *M vs. H* plots (*M* being the magnetisation per Co^II^ unit and *H* the applied dc magnetic field) for **DCB@1**, **TAN@1**, **TOL@1** and **PYR@1** (measured as solvent suspensions) were registered between 2 and 10 K and they are shown in Fig. S15.† Usually, the axial (*D*) and rhombic (*E*/*D*) ZFS parameters can be estimated from the thermal dependence of the magnetisation isotherms. Thus, the *M vs. H*/*T* plots are quite different and, when the weak magnetic couplings are ruled out, this is clear proof of the presence of non-negligible ZFS. However, when |*D*| is very large, at these low temperatures only the ground Kramer doublet is populated and the *M vs. H*/*T* plots superimpose which is the case for **DCB@1**, **TAN@1**, **TOL@1** and **PYR@1** (Fig. S16† confirms this point for **PYR@1**). In such a case, it is not possible to extract the ZFS parameters from magnetization curves, but they can be simulated by using the spin Hamiltonian for an effective doublet spin state

2 (*S*_eff_ = 1/2), *H* = *βH*(*g*_*x*_*S*_*x*_ + *g*_*y*_*S*_*y*_ + *g*_*z*_*S*_*z*_).

For **DCB@1** and **TAN@1**, simulations of the magnetisation curves were done from the components of the *g*-factor obtained from the HFEPR experiments (Table 2), which superimpose well with the experimental data (solid line in Fig. S15a and b†). Otherwise, the fits of the magnetization curves were done through the VPMAG program^48^ for **TOL@1** and **PYR@1**, where a complete HFEPR study was not possible (solid line in Fig. S15c and d†). In the last two cases, the found *g*_i_ components values are in good agreement with a positive *D* value (Table 2). We like to stress that, from the *g*_i_ and *E*/*D* values for a *S* = 3/2 of **DCB@1** (Table 2) and through the equations proposed by Gatteschi *et al.*,^50^ it is possible to evaluate the *g*_i_ components of the ground state Kramer doublet (*g*_*x*_ = 4.08, *g*_*y*_ = 5.98 and *g*_*z*_ = 2.26), which perfectly matches with those found from the HFEPR spectrum (*g*_*x*_ = 4.05, *g*_*y*_ = 6.05 and *g*_*z*_ = 2.45).

In conclusion, the combination of the thermal dependence of the magnetic susceptibility and the magnetization data, HFEPR spectroscopy and the theoretical study for **DCB@1**, **TAN@1**, **TOL@1** and **PYR@1**, unambiguously confirms the large and positive axial ZFS (*D* > 0) with non-negligible rhombic ZFS (*E*/*D* ≈ 0.12) and also their dependence on the nature of the guest molecule, which provokes non-negligible changes in the geometry of the coordination sphere of the Co^II^ ion.

### Dynamic (ac) magnetic properties: guest-induced switching of the slow magnetic relaxation

The large and positive magnetic anisotropy values observed for the octahedral cobalt(ii) ions in **DCB@1**, **TAN@1**, **TOL@1** and **PYR@1** together with the fact that Co^II^ ions are very well isolated (see the structural section), strongly suggest that slow magnetic relaxation effects typical of SIMs could be observed, prompting us to study the dynamic magnetic properties of this series of compounds.

**Fig. 5:**
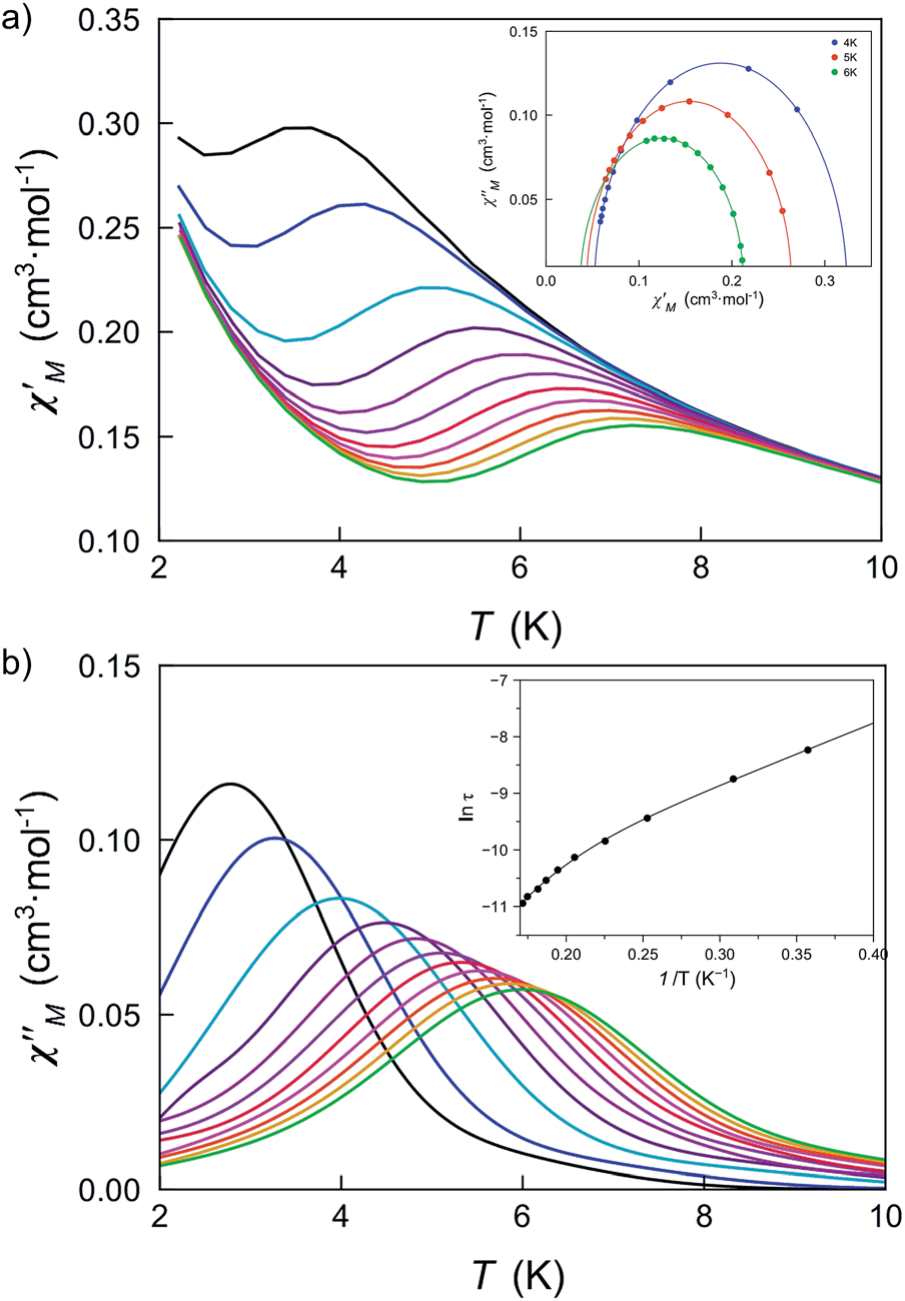
Temperature dependence of *χ*_M_′ (a) and *χ*_M_′′ (b) of **DCB@1** in a 250 G applied static field and under a ±5.0 G oscillating field in the frequency range of 0.1–10 kHz. The insets show the Cole–Cole plots at 4.0, 5.0 and 6.0 K (a) and the Arrhenius plot (b) in the high temperature region. The solid lines are the best fit curves (see Table S3†).

Firstly, we investigated the alternating current (ac) magnetic susceptibility of the parent compound **DCB@1** in the form of the *χ*_M_′ and *χ*_M_′′ *versus T* plots (*χ*_M_′ and *χ*_M_′′ being the in-phase and out-of-phase ac magnetic susceptibilities per mononuclear unit) at different applied static fields in the range of 0–1.0 kG (Fig. 5 and S17–19†). In a zero dc magnetic field, no *χ*_M_′′ signals can be observed (Fig. S17†) even for the highest frequency used (*ν* = 10 kHz), suggesting that fast zero-field quantum tunnelling relaxation of the magnetization effects are present. However, when such a small static dc field as 250 G is applied, strong frequency-dependent maxima appear in both *χ*_M_′ and *χ*_M_′′ below 10 K (Fig. 5). Additional ac measurements for **DCB@1** under higher applied dc fields of 500 and 1000 G are shown in Fig. S18 and S19,† showing the same single strong frequency-dependent *χ*_M_′′ maxima below 10 K. Interestingly, no divergence in *χ*_M_′′ below the blocking temperature (*T*_B_) was observed, even with such a very small applied dc field as 250 G.

These results are in contrast to those found in most octahedral cobalt(ii) SIMs where higher applied fields are required to observe frequency dependent signals with clear out-of-phase peaks.^22^ This observation strongly suggests that organising SIMs in a MOF is a good strategy to isolate them, thus minimising intermolecular interactions, which are often associated with complex magnetic relaxation processes.

Taking into account the slow magnetic relaxation effects observed for the parent compound of the series **DCB@1** and, considering that the exchange of the **DCB** guest molecules by **TAN**, **TOL** or **PYR** is accompanied by non-negligible distortion of the cobalt(ii) environments and tuning of the corresponding *D* values (see Table 1), it was easy to envisage that the guest exchange process can modify the dynamic magnetic properties of this series. Thus, alternating current (ac) magnetic susceptibility measurements were also carried out for **TAN@1**, **TOL@1** and **PYR@1**.

**Fig. 6:**
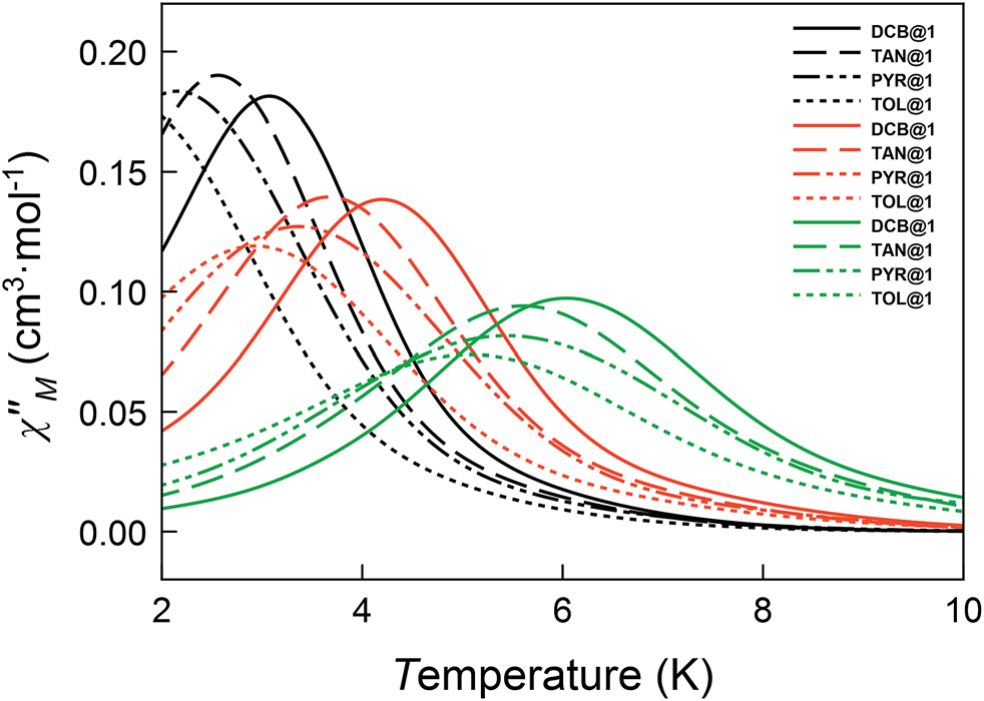
Temperature dependence of *χ*_M_′′ of **DCB@1**, **TAN@1**, **TOL@1** and **PYR@1** in a dc applied static field of 1000 G and under a ±5.0 G oscillating field at 672 (black), 2043 (orange) and 10 000 Hz (green) frequencies.

The magnetic ac measurements in a 1000 G applied static field showed frequency-dependent maxima, in both *χ*_M_′ and *χ*_M_′′, for the three adsorbates below 10 K (Fig. S20–S22†), which are similar to those observed for compound **DCB@1**. Indeed, the position of the out-of-phase (*χ*_M_′′) peaks depends on the nature of the solvent molecule hosted in the porous structure, that is, the *T*_B_ can be tuned by systematically varying the nature of the guest molecule (Fig. S19–S22†). This empirical observation is in the line of that predicted by the theoretical calculations and the experimental static magnetic properties and, ultimately confirms that the distortions of the metal environments caused by the exchange of the guest molecules also modify the dynamic magnetic properties of the cobalt(ii) centres. Fig. 6 shows in detail the switch of the *T*_B_ values at a given frequency depending on the solvent nature. In this respect, a temperature shift of up to 1.5 K is observed between **DCB@1** (highest *T*_B_) and **TOL@1** (lowest *T*_B_). The important distortions in the octahedral environments of cobalt(ii) ions (see Fig. 1 and Table S2†), caused by the exchange of the aromatic solvent guests, lie undoubtedly at the origin of this switching behaviour, as extracted from the analysis of the combined dynamic and static magnetic properties.

The Cole–Cole plots at different temperatures and applied dc fields for **DCB@1**, **TAN@1**, **TOL@1** and **PYR@1** gave almost perfect semicircles which can be fitted by the generalized Debye model^51^ (solid lines in the insets of Fig. 5a and S18a–S22a†). The calculated low values of the *α* parameter at the different applied dc fields (see Table S3†) support single relaxation processes in all the cases and discard thus a spin-glass behavior^52^ (*α* = 0 for a Debye model). Yet, for all four compounds, two different relaxation processes are observed, where the relaxation times calculated from the maximum of *χ*_M_′′ at a given frequency (*τ* = 1/2π*ν*) follow the Arrhenius law characteristic of a thermally activated mechanism for each relaxation [*τ* = ((1/*τ*_01_)exp(*E*_a1_/*k*_B_*T*) + (1/*τ*_02_)exp(*E*_a2_/*k*_B_*T*))^–1^] (solid lines in the insets of Fig. 5b and S18b–S22b†). Thus, the calculated values of the first pre-exponential factor (*τ*_0_) and activation energy (*E*_a_) (see Table S3†) are consistent with those found for previously reported octahedral cobalt(ii) SIMs.^22^,^49^ However, lower values of *E*_a_ are found for the second relaxation process.

These two different processes found in all four compounds correspond to a unique Co^II^ ion as Cole–Cole plots suggest, *i.e.*, both processes are competing in each magnetic centre. This kind of non-linear Arrhenius plot is usually attributed to the sum of different relaxation processes that include Orbach mechanism, quantum tunnelling, direct and Raman processes. For the last two, the spin-lattice relaxation time is given for *τ*^–1^ = *AT*^*n*^, where *n* = 1 for the direct mechanism and *n* = 7 or 9 for the Raman process in non-Kramer or Kramer ions, respectively. But, if optical and/or acoustic phonons are also considered, *n* values between 1 and 6 can be also reasonable. In our cases, considering only Orbach and Raman relaxation processes is enough to correctly simulate the experimental data and, therefore, the inclusion of other relaxation processes is neither needed nor have physical meaning; however, the best fits lead to *n* values around 16, which is not acceptable. Even more, correct fits can also be reached with *n* values into the range from 1 to 55.

A possible alternative consists of verifying that the {*τ*,*T*} pair follows a power law, where a linear dependence must be observed in ln(*τ*) *vs.* ln(*T*) plots. In the four studied systems and at an applied magnetic field of 1.0 kG, the *n* values fluctuate from 2 to 4 (Table S4†). However, the experimental data in both relaxation processes – Orbach and Raman – follow the same law despite of their different nature (Fig. S23†). That is why we think global relaxation involving two different Orbach processes could be more adequate to explain the SIM behaviour of this family of compounds, but an easy explanation is not evident. Anyway, taking into account the important asymmetry between the axial and perpendicular *g*-factor of Co^II^ ions, two different relaxation times (parallel and transverse) could be envisaged. On the other hand, the double Arrhenius regimes are not observed in all known Co^II^ SIMs,^53^,^54^ which could be related to different weight of the rhombic ZFS in them. However, deeper studies are necessary to confirm or reject these possibilities in most SIMs.

## Conclusions

In summary, we report a new coordination polymer (**DCB@1**) displaying single-ion magnetic behaviour, which has been constructed by following a rational synthetic strategy consisting of the use of a very long organic spacer that affords a two-dimensional structure and prevents any magnetic interaction between the cobalt centres. As a consequence, the resulting magnetically isolated, tetragonally distorted octahedral cobalt(ii) ions, exhibit slow magnetic relaxation effects typical of SIMs. This novel porous material is able to exchange, in a post-synthetic SC to SC process, the DCB molecules hosted in its pores with other aromatic molecules (TAN, TOL, and PYR) to yield three new adsorbates (**TAN@1**, **TOL@1** and **PYR@1**). These compounds also show SIM behaviour, which is dependent on the nature of the guest molecule. The exchange process, followed by single-crystal X-ray diffraction, clearly outlines a putative role of solvent guest molecules by inducing “non-innocent” distortions on the ligand conformation and, ultimately, leaving a “fingerprint” on the cobalt environments. The combined analysis of experimental (spectroscopic and magnetic properties) and theoretical (NEVPT2 calculations) data supports unambiguously that the distortions in the metal environments are responsible for this guest-dependent SIM behaviour. Overall, these results reaffirm the recent findings shown by Long *et al.*^44^ and expand the very limited scope of guest-dependent SIM-MOFs.

## Supplementary Material

Supplementary information

Crystal structure data

## References

[cit1] Sessoli R., Gatteschi D., Caneschi A., Novak M. A. (1993). Nature.

[cit2] Sessoli R., Tsai H. L., Schake A. R., Wang S., Vincent J. B., Folting K., Gatteschi D., Christou G., Hendrickson D. N. (1993). J. Am. Chem. Soc..

[cit3] Bogani L., Wernsdorfer W. (2008). Nat. Mater..

[cit4] Sanvito S. (2011). Chem. Soc. Rev..

[cit5] Kahn O., Molecular Magnetism, VCH Publishers, New York, 1993.

[cit6] Maspoch D., Ruiz-Molina D., Veciana J. (2007). Chem. Soc. Rev..

[cit7] Dechambenoit P., Long J. R. (2011). Chem. Soc. Rev..

[cit8] Grancha T., Ferrando-Soria J., Castellano M., Julve M., Pasán J., Armentano D., Pardo E. (2014). Chem. Commun..

[cit9] Ishikawa N., Sugita M., Ishikawa T., Koshihara S.-Y., Kaizu Y. (2003). J. Am. Chem. Soc..

[cit10] Ishikawa N., Sugita M., Ishikawa T., Koshihara S., Kaizu Y. (2004). J. Phys. Chem. B.

[cit11] Ishikawa N., Sugita M., Wernsdorfer W. (2005). J. Am. Chem. Soc..

[cit12] Yamashita A., Watanabe A., Akine S., Nabeshima T., Nakano M., Yamamura T., Kajiwara T. (2011). Angew. Chem..

[cit13] Zhang P., Zhang L., Wang C., Xue S., Lin S.-Y., Tang J. (2014). J. Am. Chem. Soc..

[cit14] Rinehart J. D., Long J. R. (2009). J. Am. Chem. Soc..

[cit15] Rinehart J. D., Meihaus K. R., Long J. R. (2010). J. Am. Chem. Soc..

[cit16] Mougel V., Chatelain L., Pécaut J., Caciuffo R., Colineau E., Griveau J.-C., Mazzanti M. (2012). Nat. Chem..

[cit17] Magnani N., Colineau E., Griveau J.-C., Apostolidis C., Walter O., Caciuffo R. (2014). Chem. Commun..

[cit18] Harman W. H., Harris T. D., Freedman D. E., Fong H., Chang A., Rinehart J. D., Ozarowski A., Sougrati M. T., Grandjean F., Long G. J., Long J. R., Chang C. J. (2010). J. Am. Chem. Soc..

[cit19] Freedman D. E., Harman W. H., Harris T. D., Long G. J., Chang C. J., Long J. R. (2010). J. Am. Chem. Soc..

[cit20] Jurca T., Farghal A., Lin P., Korobkov I., Murugesu M., Richeson D. S. (2011). J. Am. Chem. Soc..

[cit21] Zadrozny J. M., Long J. R. (2011). J. Am. Chem. Soc..

[cit22] Vallejo J., Castro I., Ruiz-García R., Cano J., Julve M., Lloret F., de Munno G., Wernsdorfer W., Pardo E. (2012). J. Am. Chem. Soc..

[cit23] Zadrozny J. M., Atanasov M., Bryan A. M., Lin C.-Y., Rekken B. D., Power P. P., Neese F., Long J. R. (2013). Chem. Sci..

[cit24] Vallejo J., Pascual-Álvarez A., Cano J., Castro I., Julve M., Lloret F., Krzystek J., de Munno G., Armentano D., Wernsdorfer W., Ruiz-García R., Pardo E. (2013). Angew. Chem., Int. Ed..

[cit25] Chen L., Wang J., Wei J.-M., Wernsdorfer W., Chen X.-T., Zhang Y.-Q., Song Y., Xue Z.-L. (2014). J. Am. Chem. Soc..

[cit26] Martínez-Lillo J., Mastropietro T. F., Lhotel E., Paulsen C., Cano J., de Munno G., Faus J., Lloret F., Julve M., Nellutla S., Krzystek J. (2013). J. Am. Chem. Soc..

[cit27] Bradshaw D., Claridge J. B., Cussen E. J., Prior T. J., Rosseinsky M. J. (2005). Acc. Chem. Res..

[cit28] Férey G. (2008). Chem. Soc. Rev..

[cit29] Janiak C. (2003). Dalton Trans..

[cit30] Kitagawa S., Matsuda R. (2007). Coord. Chem. Rev..

[cit31] Long J. R., Yaghi O. M. (2009). Chem. Soc. Rev..

[cit32] Rosi N. L., Eddaoudi M., Kim J., O'Keeffe M., Yaghi O. M. (2002). CrystEngComm.

[cit33] Halder G. J., Kepert C. J., Moubaraki B., Murray K. S., Cashion J. D. (2002). Science.

[cit34] Agusti G., Ohtani R., Yoneda K., Gaspar A. B., Ohba M., Sánchez-Royo J. F., Muñoz M. C., Kitagawa S., Real J. A. (2009). Angew. Chem., Int. Ed..

[cit35] Ohba M., Yoneda K., Agusti G., Muñoz M. C., Gaspar A. B., Real J. A., Yamasaki M., Ando H., Nakao Y., Sakaki S., Kitagawa S. (2009). Angew. Chem., Int. Ed..

[cit36] Yaghi O. M., O'Keeffe M., Ockwig N. W., Chae H. K., Eddaoudi M., Kim J. (2003). Nature.

[cit37] Li J.-R., Kuppler R. J., Zhou H.-C. (2009). Chem. Soc. Rev..

[cit38] Ferrando-Soria J., Khajavi H., Serra-Crespo P., Gascon J., Kapteijn F., Julve M., Lloret F., Pasán J., Ruiz-Pérez C., Journaux Y., Pardo E. (2012). Adv. Mater..

[cit39] Baldoví J. J., Coronado E., Gaita-Ariño A., Gamer C., Giménez-Marqués M., Mínguez Espallargas G. (2014). Chem.–Eur. J..

[cit40] Champness N. R., Khlobystov A. N., Majuga A. G., Schröder M., Zyk N. V. (1999). Tetrahedron Lett..

[cit41] Ion A. E., Nica S., Madalan A. M., Shova S., Vallejo J., Julve M., Lloret F., Andruh M. (2015). Inorg. Chem..

[cit42] Palion-Gazda J., Klemens T., Machura B., Vallejo J., Lloret F., Julve M. (2015). Dalton Trans..

[cit43] Liu X., Sun L., Zhou H., Cen P., Jin X., Xie G., Chen S., Hu Q. (2015). Inorg. Chem..

[cit44] Zhang X., Vieru V., Feng X., Liu J.-L., Zhang Z., Na B., Shi W., Wang B.-W., Powell A. K., Chibotaru L. F., Gao S., Cheng P., Long J. R. (2015). Angew. Chem., Int. Ed..

[cit45] Real J. A., Andrés E., Muñoz M. C., Julve M., Granier T., Bousseksou A., Varret F. (1995). Science.

[cit46] Moliner N., Muñoz M. C., Letard S., Solans X., Menendez N., Goujon A., Varret F., Real J. A. (2000). Inorg. Chem..

[cit47] Lloret F., Julve M., Cano J., Ruiz-García R., Pardo E. (2008). Inorg. Chim. Acta.

[cit48] Cano J., VPMAG package, University of Valencia, Valencia, Spain, 2003.

[cit49] Colacio E., Ruiz J., Ruiz E., Cremades E., Krzystek J., Carretta S., Cano J., Guidi T., Wernsdorfer W., Brechin E. K. (2013). Angew. Chem., Int. Ed..

[cit50] Banci L., Bencini A., Benelli C., Gatteschi D., Zanchini C. (1982). Struct. Bonding.

[cit51] Cole R. H., Cole K. S. (1941). J. Chem. Soc..

[cit52] Mydosh J. A., Spin Glasses: An Experimental Introduction, Taylor & Francis, London, 1993.

[cit53] Yang F., Zhou Q., Zhang Y., Zeng G., Li G., Shi Z., Wang B., Feng S. (2013). Chem. Commun..

[cit54] Wu D., Zhang X., Huang P., Huang W., Ruan M., Ouyang Z. W. (2013). Inorg. Chem..

