# Supplementary material for: Guest-dependent single-ion magnet behaviour in a cobalt(ii) metal–organic framework
Source: Chem Sci. 2015 Dec 10;7(3):2286–93. doi: 10.1039/c5sc04461h (PMC5977934; doi:10.1039/c5sc04461h)
Supplement: Supplementary file 1 [file SC-007-C5SC04461H-s001.pdf]

Electronic Supplementary Material (ESI) for Chemical Science.
This journal is © The Royal Society of Chemistry 2015

Electronic Supporting Information (ESI) for the manuscript:

Guest-Dependent Single-Ion Magnet Behaviour in a Cobalt(II) Metal-Organic Framework

Julia Vallejo, Francisco R. Fortea-Pérez, Emilio Pardo,* Samia Benmansour, Isabel Castro, J. Krzystek, Donatella Armentano and Joan Cano*

Experimental Section

Materials. All chemicals were of reagent grade quality, and they were purchased from commercial sources and used as received.

1,4-bis(4'-Pyridylethynyl)benzene(bpeb). The bpeb ligand was synthesized by using a slightly modified previously reported procedure.[1] The only difference can be found in the final step where, instead of recrystallizing the residue from toluene, it was purified by thoroughly washing with cold water and ether. The product was isolated as a yellow solid. Yield 99%. IR (KBr): 2220 ($\nu_{C\equiv C}$). ^{1}H NMR ($CDCl_3$): 7.41 (4H, d), 7.58 (4H, s), 8.64 (4H, d). ^{13}C NMR ($CDCI_3$): 88.6, 93.2 (C≡C), 123.0, 125.5, 131.2, 132.0, 149.9.

Preparation of [Co(bpeb)$_2$(NCS)$_2$]·7DCB (DCB@1). Well-formed orange cubic prisms of **DCB@1**, suitable for X-ray diffraction, were grown after a few days of slow layer diffusion in an essay tube at room temperature. The top layer was a methanol solution of the Co^{II}(NCS)2 (0.0088 g, 0.05 mmol), while the bottom one was a dichlorobenzene/methanol solution (4:1 v/v) of the bpeb ligand (0.028 g, 0.1 mmol). The crystals were collected by filtration and air-dried (0.040 g, 42% yield); elemental analysis calculated (%) for $C_{84}H_{52}Cl_{14}CoN_6S_2$ (1756.9): C 57.17, H 2.97, N 4.76, S 3.63; found: C 56.96, H 3.11, N 4.56, S 3.69. IR (KBr) 2080((ν_{NCS}), 1497, 1590 ($\nu_{C=C}$) and 2218 cm–1 ($\nu_{C\equiv C}$).

Preparations of [Co(bpeb)$_2$(NCS)$_2$]·4TAN·4MeOH (TAN@1), [Co(bpeb)$_2$(NCS)$_2$]·6TOL (TOL@1) and [Co(bpeb)$_2$(NCS)$_2$]·8PYR (PYR@1). Well-formed orange cubic prism of **TAN@1**, **TOL@1** and **PYR@1**, which were suitable for X-ray diffraction, were obtained by immersing crystals of **DCB@1** for a week in thianthrene, toluene and pyrrole solutions, respectively. The crystals were collected by filtration and air-dried. **TAN@1**: elemental analysis calculated (%) for $C_{94}H_{72}CoN_6\ O_4S_{10}$ (1727.2): C 65.29, H 4.20, N 4.86, S18.54; found: C 64.98, H 4.35, N 4.90, S 18.60, IR (KBr) 2080(ν_{NCS}), 1462, 1495, 1590 ($\nu_{C=C}$) and 2219 cm–1 ($\nu_{C\equiv C}$). **TOL@1**: elemental analysis calculated (%) for $C_{84}H_{72}CoN_6S_2$ (1287.5): C 78.30, H 5.63, N 6.52, S 4.98; found: C 78.36, H 5.85, N 6.47, S 4.97. IR (KBr) 2081(ν_{NCS}), 3087, 3062(ν_{CH3}), 1497, 1590($\nu_{C=C}$) and 2218 cm–1 ($\nu_{C\equiv C}$). **PYR@1**: elemental analysis calculated (%) for $C_{74}H_{64}CoN_{14}S_2$ (1271.4): C 69.85, H 5.07, N 15.41, S 5.04; found: C 69.97, H 4.96, N 15.46, S 5.01. IR (KBr) 2080(ν_{NCS}), 3400(ν_{NH}) 1494, 1589 ($\nu_{C=C}$) and 2218 cm–1 ($\nu_{C\equiv C}$).

[1] Champness, N. R.; Khlobystov, A. N.; Majuga, A. G.; Schröder, M.; Zyk, N. V. *Tetrahedron Lett.* **1999**, *40* (29), 5413.

Physical Techniques. Elemental (C, H, S, N) and Inductively coupled plasma–atomic emission spectroscopy (ICP-AES) analyses were performed at the Microanalytical Service of the Universitat de València.

Thermogravimetric Analysis. The thermogravimetric analysis (TGA) was performed on crystalline samples of **DCB@1**, **TAN@1**, **TOL@1** and **PYR@1** under a dry N_2 atmosphere with a Mettler Toledo TGA/STDA 851e thermobalance operating at a heating rate of 10 ºC min$^{–1}$.

X-ray Powder Diffraction. Polycrystalline samples of **DCB@1**, **TAN@1**, **TOL@1** and **PYR@1** were introduced into 0.5 mm borosilicate capillaries prior to being mounted and aligned on a Empyrean PANalytical powder diffractometer, using Cu Kα radiation (λ = 1.54056 Å). For each sample, three repeated measurements were collected at 100 K (2θ = 2–40°) and merged in a single diffractogram.

HFEPR Measurements. HFEPR spectra of **DCB@1**, **TAN@1**, **TOL@1** and **PYR@1** were recorded at 4.5 K on polycrystalline samples (20-25 mg) suspended in the corresponding solvent (see experimental section) to prevent any possible desolvation, using a homodyne spectrometer associated with a 15/17-T superconducting magnet. The presence of solvent strongly restricted the frequency to values lower than ~ 200 GHz. Detection was provided with an InSb hot electron bolometer (QMC Ltd., Cardiff, UK). The magnetic field was modulated at 50 kHz for detection purposes. A Stanford Research Systems SR830 lock-in amplifier converted the modulated signal to dc voltage. The single-frequency spectra were simulated with the SPIN software.

Crystal Structure Data Collection and Refinement. Crystal data for **DCB@1**: $C_{84}H_{52}Cl_{14}CoN_6S_2$, triclinic, space group *P*(-1), *a* = 13.593(5) Å, *b* = 17.194(5) Å, *c* = 18.450(5) Å, α = 99.319(5)°, β = 100.076(5)°, γ = 107.842(5)° V = 3933(2) Å^3, T = 100(2) K, Z = 2, ρ_{calc} = 1.490 g.cm^{-3}, μ = 0.797 mm^{-1}, of the 59588 reflections collected (R_{int} = 0.0526), 8262 are unique and 5878 observed with $I > 2\sigma(I)$. Refinement of 386 parameters gave R = 0.1485 and R_w = 0.3876 for reflections with $I > 2\sigma(I)$ and R = 0.1824 and R_w = 0.4104 for all reflections, with S = 1.483. **TAN@1**: $C_{94}H_{72}CoN_6O_4S_{10}$ monoclinic, space group *C*2/*m*, *a* = 13.8432(9) Å, *b* = 20.8218(9) Å, *c* = 14.0803(6) Å, β = 96.987(3)°, V = 4028.4(3) Å^3, T = 100(2) K, Z = 2, ρ_{calc} = 1.426 g.cm^{-3}, μ = 0.531 mm^{-1}, of the 34324 reflections collected (R_{int} = 0.0268), 2689 are unique and 2472 observed with $I > 2\sigma(I)$. Refinement of 141 parameters gave R = 0.0697 and R_w = 0.2019 for reflections with $I > 2\sigma(I)$ and R = 0.0738 and R_w = 0.2056 for all reflections, with S = 1.073. **TOL@1**: $C_{84}H_{72}CoN_6S_2$, monoclinic, space group *C*2/*c*, *a* = 31.601(4) Å, *b* = 21.039(4)

Å, c = 27.275(5) Å, β = 121.629(11)°, V = 15440(5) Å^3, T = 100(2) K, Z = 8, ρ_{calc} = 1.109 g.cm^{-3}, μ = 0.321 mm^{-1}, of the 55448 reflections collected (R_{int} = 0.0892), 13092 are unique and 5828 observed with $I > 2\sigma(I)$. Refinement of 467 parameters gave R = 0.0872 and R_w = 0.2189 for reflections with $I > 2\sigma(I)$ and R = 0.1581 and R_w = 0.2372 for all reflections, with S = 1.054. **PYR@1**: $C_{74}H_{64}CoN_{14}S_2$, triclinic, space group P(-1), a = 11.653(4) Å, b = 13.567(6) Å, c = 13.662(6) Å, α = 94.50(2)°, β = 94.78(2)°, γ = 111.12(2)° V = 1994.0(14) Å^3, T = 100(2) K, Z = 1,, ρ_{calc} = 1.060 g.cm^{-3}, μ = 0.313 mm^{-1}, of the 11175 reflections collected (R_{int} = 0.0883), 4058 are unique and 2223 observed with $I > 2\sigma(I)$. Refinement of 143 parameters gave R = 0.1702 and R_w = 0.3830 for reflections with $I > 2\sigma(I)$ and R = 0.2098 and R_w = 0.4078 for all reflections, with S = 1.886.

Single crystals of **DCB@1**, **TAN@1**, **TOL@1** and **PYR@1** were selected and mounted on a MITIGEN holder in Paratone oil and very quickly placed on a liquid nitrogen stream cooled at 100 K to avoid the possible degradation upon desolvation. Diffraction data were collected on a Bruker-Nonius X8APEXII CCD area detector diffractometer using graphite-monochromated Mo-K_α radiation (λ = 0.71073 Å). As reported, crystals of **DCB@1**, **TAN@1**, **TOL@1** and **PYR@1**, suitable for X-ray diffraction, were obtained by immersing crystals of **DCB@1** for a week in thianthrene, toluene and pyrrole solutions, respectively, after a crystal-to-crystal transformation accounting for a poor quality and a poor diffraction power of the samples. In fact, a lower θ_{max} of diffraction were obtained, especially for **PYR@1**, even if all possible steps were undertaken to ensure that the experiment was able to extract the best diffracting power from the sample. However, since the solution and refinement parameters are reasonable, compared with analogue 2D MOFs structures previously reported, we are confident that the crystal structure found is consistent. The data were processed through the SAINT[2] reduction and SADABS[3] multi-scan absorption software. The structure was solved with the ShelXS structure solution program, using the Direct Methods solution method. The model was refined with version 2013/4 of ShelXL against F^2 on all data by full-matrix least squares.[4] All non-hydrogen atoms were refined anisotropically. The hydrogen atoms of the organic ligands were set on geometrical positions and refined with a riding model. Keeping in mind that we are dealing with a SC to SC solid-state incorporation of the solvent guests into the 2D networks, it is not surprising that the thianthrene, toluene and pyrrole molecules found from ΔF map were disordered within the voids of the structure. In particular, no good model for

[2] SAINT, version 6.45, Bruker Analytical X-ray Systems, Madison, WI, 2003.
[3] Sheldrick G.M. SADABS Program for Absorption Correction, version 2.10, Analytical X-ray Systems, Madison, WI, 2003
[4] (a) G. M. Sheldrick, *Acta Cryst.* **2008**, *A64*, 112-122. (b) SHELXTL-2013/4, Bruker Analytical X-ray Instruments, Madison, WI, 2013.

guest molecules in **PYR@1** has been found. Residual electron densities in the solvent-accessible void due to disordered solvent molecules were treated with the PLATON SQUEEZE program.[5] Crystal data before the treatment of SQUEEZE program for **DCB@1**: Refinement of 659 parameters gave $R = 0.2181$ and $R_w = 0.5409$ for reflections with $I > 2\sigma(I)$ and $R = 0.2422$ and $R_w = 0.5584$ for all reflections, with $S = 2.434$. **TAN@1**: Refinement of 172 parameters gave $R = 0.1372$ and $R_w = 0.3916$ for reflections with $I > 2\sigma(I)$ and $R = 0.1426$ and $R_w = 0.4001$ for all reflections, with $S = 1.572$. **TOL@1**: Refinement of 550 parameters gave $R = 0.1359$ and $R_w = 0.3739$ for reflections with $I > 2\sigma(I)$ and $R = 0.2071$ and $R_w = 0.4006$ for all reflections, with $S = 1.207$. **PYR@1**: Refinement of 179 parameters gave $R = 0.3424$ and $R_w = 0.6591$ for reflections with $I > 2\sigma(I)$ and $R = 0.4123$ and $R_w = 0.6918$ for all reflections, with $S = 4.110$.

Both CIFs for **DCB@1, TAN@1** and **TOL@1** before and after the treatment of SQUEEZE program have been deposited at Cambridge Structural Database, CCDC 1415915-1415921. The final geometrical calculations on free voids and the graphical manipulations were carried out with PLATON[6] implemented in WinGX[7], and CRYSTAL MAKER[8], respectively.

Magnetic Measurements. Variable-temperature (2.0–300 K) direct current (dc) magnetic susceptibility measurements under an applied field of 100 G ($T < 30$ K) and 1000 G ($T \geq 20$ K), and variable-field (0–5.0 T) magnetization measurements at low temperatures in the range of 2.0–10.0 K were carried out for **DCB@1** with a Quantum Design SQUID magnetometer. Variable-temperature (2.0–10 K) alternating current (ac) magnetic susceptibility measurements were carried out for **DCB@1, TAN@1, TOL@1** and **PYR@1** with a Quantum Design Physical Property Measurement System (PPMS). Static dc magnetic measurements were carried out for **DCB@1** by powdering and restraining the sample in order to prevent any displacement due to its magnetic anisotropy, whereas the dynamic ac magnetic measurements were carried out by using frozen solutions of polycrystalline samples of **DCB@1**, **TAN@1**, **TOL@1** and **PYR@1** in the respective solvent. The susceptibility data were corrected for the diamagnetism of both the constituent atoms

---

[5] (a) A. L. Spek, *Acta Crystallogr., Sect. A: Fundam. Crystallogr.*, **1990**, *46*, C34; (b) A. L. Spek, *Acta Crystallogr. D. Biol. Crystallogr.* **2009**, *65*, 148–55.
[6] Spek, A. L. *Acta Crystallogr. Sect. D, Biol. Crystallogr.* **2009**, *65*, 148.
[7] Farrugia, L. J. *J. Appl. Crystallogr.* **1999**, *32*, 837.
[8] D. Palmer, CRYSTAL MAKER, Cambridge University Technical Services, C. No Title, 1996.

and the sample holder. Dynamic ac magnetic measurements were repeated several times for all samples to confirm reproducibility.

Computational details. In order to evaluate the parameters that determine the axial (*D*) and rhombic (*E*) *zfs*, calculations based on a second order N-electron valence state perturbation theory (NEVPT2) applied on a wave function, which was previously obtained from a complete active space (CAS) calculation, were performed on a mononuclear Co(II) complex extracted from the structurally characterized molecular geometry of **DCB@1**, **TAN@1**, **TOL@1** and **PYR@1**. This mononuclear species conserve the experimental dispositions of the ligands around the metal but they have been partially simplified in order to save a large amount of cpu time. However, a calculation on **DCB@1** considering full ligands was done to verify that the simplification has no influence on the results. Thus, *D* values equal to +91.2 and +90.3 cm^{-1} was found for the modelized and more complete molecules, respectively. These calculations were carried out with version 3.0 of the ORCA programme[9] using the *TZVP* basis set proposed by Ahlrichs[10] and the auxiliary TZV/C Coulomb fitting basis sets[11]. The 2nd order contributions to *zfs* from 10 quartet and 20 doublet excited states generated from an active space with seven electrons in five d orbitals were included.

---

[9] F. Neese, *Wiley Interdiscip. Rev.: Comput. Mol. Sci.* **2012**, *2*, 73.
[10] (a) A. Schafer, H. Horn, R. Ahlrichs, *J. Chem. Phys.* **1992**, *97*, 2571. (b) A. Schafer, C. Huber, R. Ahlrichs, *J. Chem. Phys.* **1994**, *100*, 5829.
[11] (a) K. Eichkorn, O. Treutler, H. Ohm, M. Haser, R. Ahlrichs, *Chem. Phys. Lett.* **1995**, *240*, 283. (b) K. Eichkorn, O. Treutler, H. Ohm, M. Haser, R. Ahlrichs, *Chem. Phys. Lett.* **1995**, *242*, 652; (c) K. Eichkorn, F. Weigend, O. Treutler, H. Ohm, R. Ahlrichs, R. *Theor. Chem. Acc.* **1997**, *97*, 19.

Table S1. Summary of Crystallographic Data for DCB@1, TAN@1, TOL@1, and PYR@1.

	**DCB@1**	**TAN@1**	**TOL@1**	**PYR@1**
formula	$C_{84}H_{52}Cl_{14}CoN_6S_2$	$C_{94}H_{72}CoN_6O_4S_{10}$	$C_{84}H_{72}CoN_6S_2$	$C_{74}H_{64}CoN_{14}S_2$
M (g mol^{-1})	1764.66	1729.10	1288.52	1272.44
crystal system	Triclinic	Monoclinic	Monoclinic	Triclinic
space group	*P*(-1)	*C*2/*m*	*C2/c*	*P*(-1)
a (Å)	13.593(5)	13.8432(9)	31.601(4)	11.653(4)
b (Å)	17.194(5)	20.8218(9)	21.039(4)	13.567(6)
c (Å)	18.450(5)	14.0803(6)	27.275(5)	13.662(6)
α (°)	99.319(5)			94.50(2)
β (°)	100.076(5)	96.987(3)°	121.629(11)	94.78(2)
γ (°)	107.842(5)°			111.12(2)
V (Å^3)	3933(2)	4028.4(3)	15440(5)	1994.0(14)
Z	2	2	8	1
ρ_{calc} (g cm^{-3})	1.490	1.426	1.109	1.060
μ (mm^{-1})	0.797	0.531	0.321	0.313
T (K)	100	100	100	100
Unique reflections	8262	2689	13092	4058
Observed reflections [$I > 2\sigma(I)$]	5878	2472	5828	2223
R^a [$I > 2\sigma(I)$] (all data)	0.1485 (0.1824)	0.0697 (0.0738)	0.0872 (0.1581)	0.1702 (0.2098)
wR^b [$I > 2\sigma(I)$] (all data)	0.3876 (0.4104)	0.2019 (0.2056)	0.2189 (0.2372)	0.3830 (0.7078)

^{a} $R = \sum(|F_o| - |F_c|)/\sum|F_o|$. ^{b} $wR = [\sum w(|F_o| - |F_c|)^2/\sum w|F_o|^2]^{1/2}$.

Table S2. Selected structural data for DCB@1, TAN@1, TOL@1, and PYR@1.[a]

	**DCB@1**	**TAN@1**	**TOL@1**	**PYR@1**
Co–N_{NCS}[b] [Å] at Co1	2.094(9)	2.056(4)	2.087(4)	2.029(10)
Co–N_{NCS}[b] [Å] at Co2	2.124(8)	/	2.070(6)	/
Co–N_{bpeb}[b] [Å] at Co1	2.240(6)	2.191(4)	2.212(4)	2.220(5)
Co–N_{bpeb}[b] [Å] at Co2	2.223(7)	/	2.227(5)	/
N–Co–N[c] [°] at Co1	90.0(3)	90.0(1)	90.0(1)	90.0(3)
N–Co–N[c] [°] at Co2	90.0(2)	/	90.0(1)	/
N–Co–N[d] [°] at Co1	180.0(3)	180.0	180.0(2)	180.0(5)
N–Co–N[d] [°] at Co2	180.0	/	178.9(2)	/
δ[e] [°]	1.1(3)	0.0	8.0(4)	36.8(2)
	1.2(3)	35.2(2)	2.2(1)	30.4(2)
	34.0(2)		35.3(2) 47.8(1)	
	31.3(2)			
φ[f] [°]	82.83(6)	90.0	86.58(5)	96.5(3)
r_1[g] [Å]	20.823(2)	20.822(2)	20.983(2)	20.828(4)
	20.915(2)	20.912(2)	21.039(2)	21.170(4)
r_2[h] [Å]	11.547(2)	12.502(2)	11.760(2)	11.653(2)

[a] The estimated standard deviations are given in parentheses. [b] Average values of bond lengths at the cobalt atoms. [c] Average values of the interbond equatorial angles at the cobalt atoms. [d] Average values of the interbond axial angles at the cobalt atoms. [e] dihedral angle between the terminal pyridine and central benzene rings. [f] Intralayer cobalt(II)-cobalt(II)-cobalt(II) angle. [g] Intralayer Co(II)···Co(II) distances. [h] Shortest interlayer Co(II)···Co(II) distances.

Table S3. Selected ac magnetic data for DCB@1, TAN@1, TOL@1, and PYR@1 at different dc applied fields.

Compound	H^a (G)	$\tau_0(1)^b \times 10^7$ (s)<br>$\tau_0(2)^b \times 10^5$ (s)	$E_a{}^b$ (cm^{-1})	α^c	$\chi_S{}^c$ (cm^3 mol^{-1})	$\chi_T{}^c$ (cm^3 mol^{-1})
**DCB@1**	250	0.23<br>0.55	29.9<br>7.6	0.033 (4K)<br>0.012 (5K)<br>0.023 (6K)	0.14 (4K)<br>0.11 (5K)<br>0.09 (6K)	0.32 (4K)<br>0.26 (5K)<br>0.21 (6K)
**DCB@1**	500	0.27<br>0.48	29.2<br>8.6	0.023 (4K)<br>0.012 (5K)<br>0.009 (6K)	0.05 (4K)<br>0.04 (5K)<br>0.04 (6K)	0.32 (4K)<br>0.26 (5K)<br>0.21 (6K)
**DCB@1**	1000	0.17<br>0.41	31.3<br>8.8	0.024 (4K)<br>0.015 (5K)<br>0.012 (6K)	0.01 (4K)<br>0.01 (5K)<br>0.01 (6K)	0.32 (4K)<br>0.26 (5K)<br>0.21 (6K)
**TAN@1**	1000	2.27<br>0.78	17.1<br>6.2	0.058 (4K)<br>0.027 (5K)<br>0.017 (6K)	0.02 (4K)<br>0.01 (5K)<br>0.01 (6K)	0.30 (4K)<br>0.24 (5K)<br>0.20 (6K)
**TOL@1**	1000	8.22<br>3.00	11.5<br>2.5	0.071 (4K)<br>0.075 (5K)<br>0.062 (5.5K)	0.03 (4K)<br>0.02 (5K)<br>0.01 (5.5K)	0.28 (4K)<br>0.23 (5K)<br>0.20 (5.5K)
**PYR@1**	1000	1.30<br>1.02	21.0<br>4.9	0.073 (4K)<br>0.124 (5K)<br>0.105 (6K)	0.03 (4K)<br>0.02 (5K)<br>0.01 (6K)	0.24 (4K)<br>0.19 (5K)<br>0.16 (6K)

[a] Applied dc magnetic field. [b] The values of the pre-exponential factor (τ_0) and activation energy (E_a) are calculated through the Arrhenius law [$\tau = ((1/\tau_{01}) \exp(E_{a1}/k_B T) + (1/\tau_{02}) \exp(E_{a2}/k_B T))^{-1}$]. [c] The values of the α parameter, adiabatic (χ_S) and isothermal (χ_T) susceptibilities are calculated from the experimental data at different temperatures through the generalized Debye law (see text).

Table S4. Exponent of a power-law probability distributions (*n*) of relaxation times (τ) with the temperature ($\tau^{-1} = CT^n$) of DCB@1, TAN@1 , TOL@1 and PYR@1 in a 1.0 kG applied static field.

Compound	*H* (G)	*n*	*C*
**DCB@1**	1000	4.07	39.9
**TAN@1**	1000	3.39	171
**TOL@1**	1000	2.66	819
**PYR@1**	1000	2.87	412

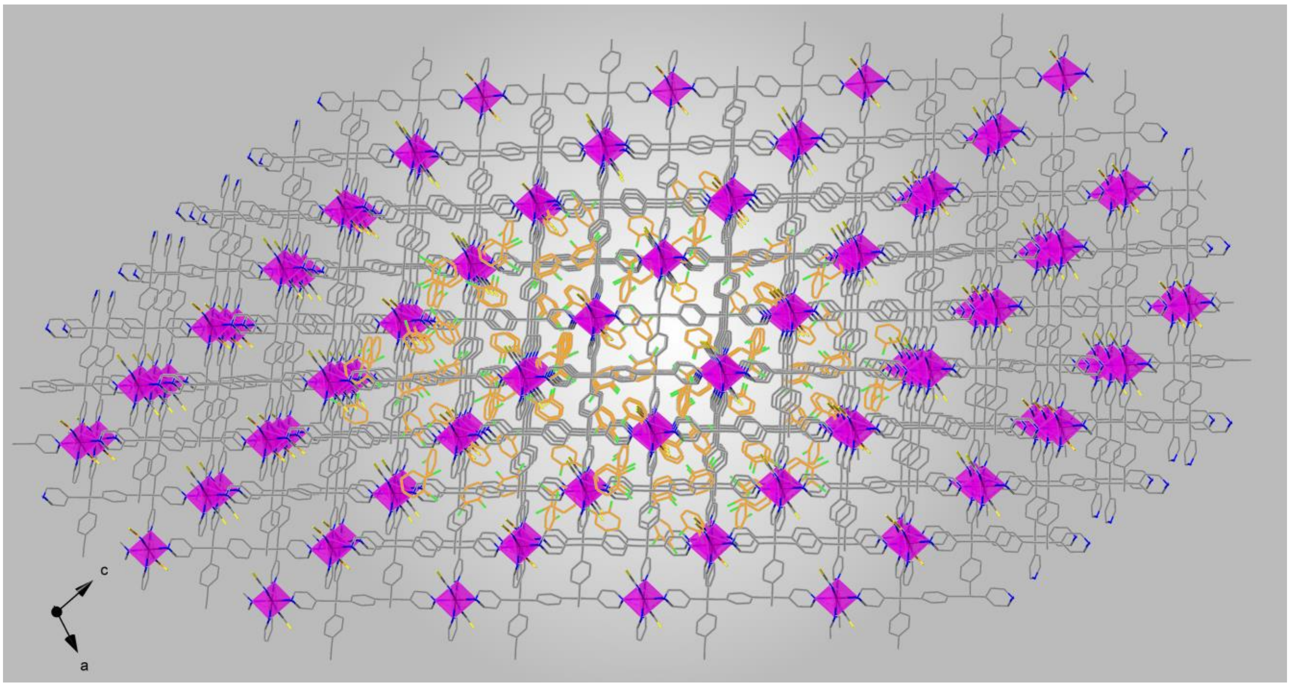

Fig. S1. Projection view of a fragment of square layers of **DCB@1** along the *b* crystallographic axis showing the filling of the pores by o-dcb cations. Cobalt atoms are represented by purple polyhedra whereas the ligands are depicted as sticks. The carbon and chlorine atoms of the guest molecules have been represented as orange and green sticks respectively and only in a portion of the 2D net for the safe of clarity.

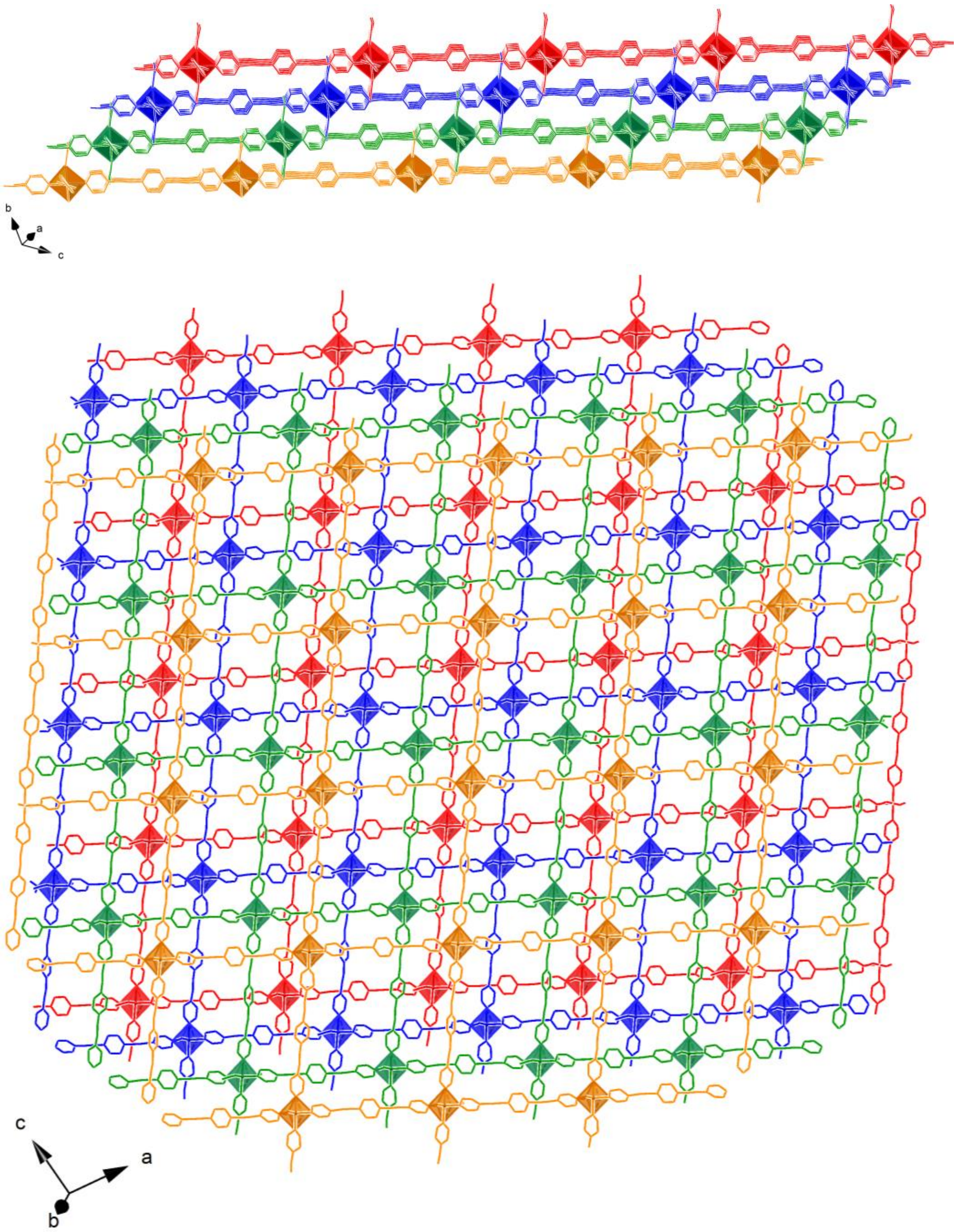

Fig. S2. Perspective views of the pilled 2D networks of **DCB@1** along the *a* crystallographic axis (top) and the direction of the normal to the plane (0 1 0) (bottom) showing an *ABCD* pattern. Cobalt atoms and ligands are represented as sticks of different colors: red (*A*), blue (*B*), green (*C*) and orange (*D*).

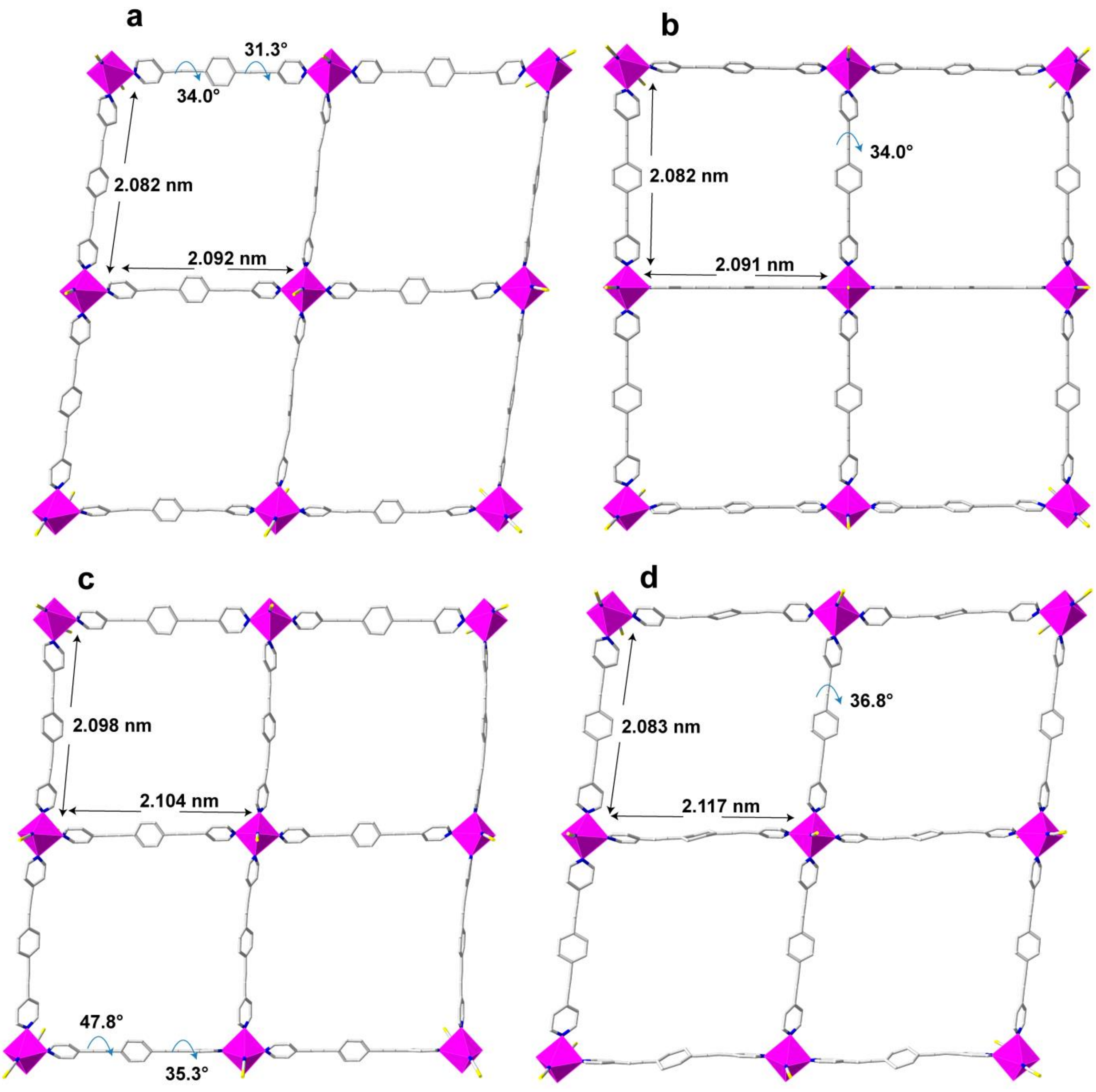

Fig. S3. Perspective view of a fragment of the neutral square grid-type flat layer of **DCB@1** (a), **TAN@1** (b), **TOL@1** (c), and **PYR@1** (d) with the side lengths and the main structural variations related to the values of the dihedral angle between the terminal pyridine and central benzene rings (δ). Cobalt atoms from the coordination network are represented by purple polyhedra whereas the ligands are depicted as sticks (hydrogen atoms are omitted for clarity).

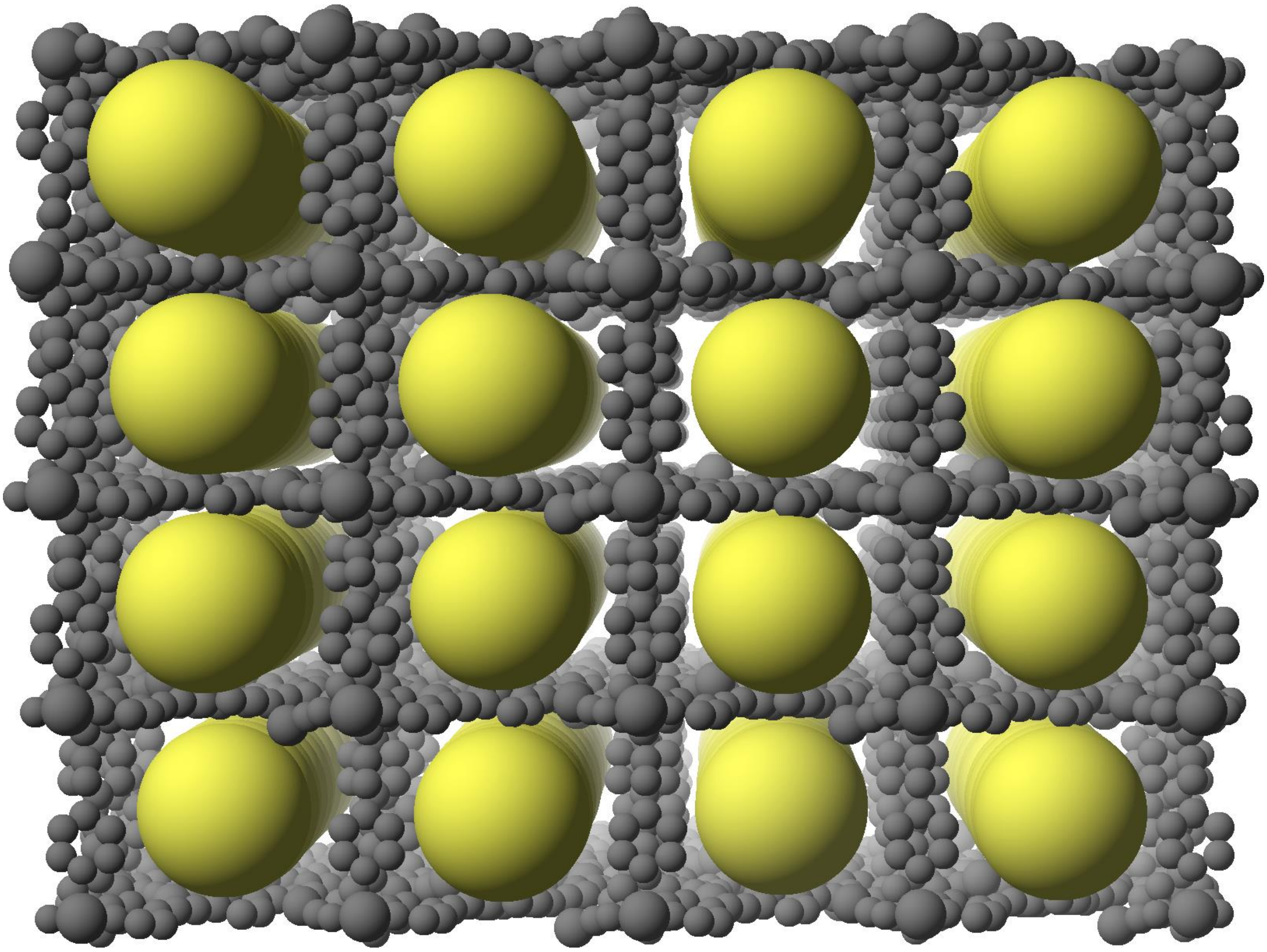

Fig. S4. Perspective view of the pilled 2D networks of **DCB@1** along the normal to (0 1 0) direction (the crystallization solvent molecules are omitted for clarity). All atoms are represented as grey space filling spheres (Van der Waals radii). The void space accessible for the guest aromatic species is represented by yellow spheres.

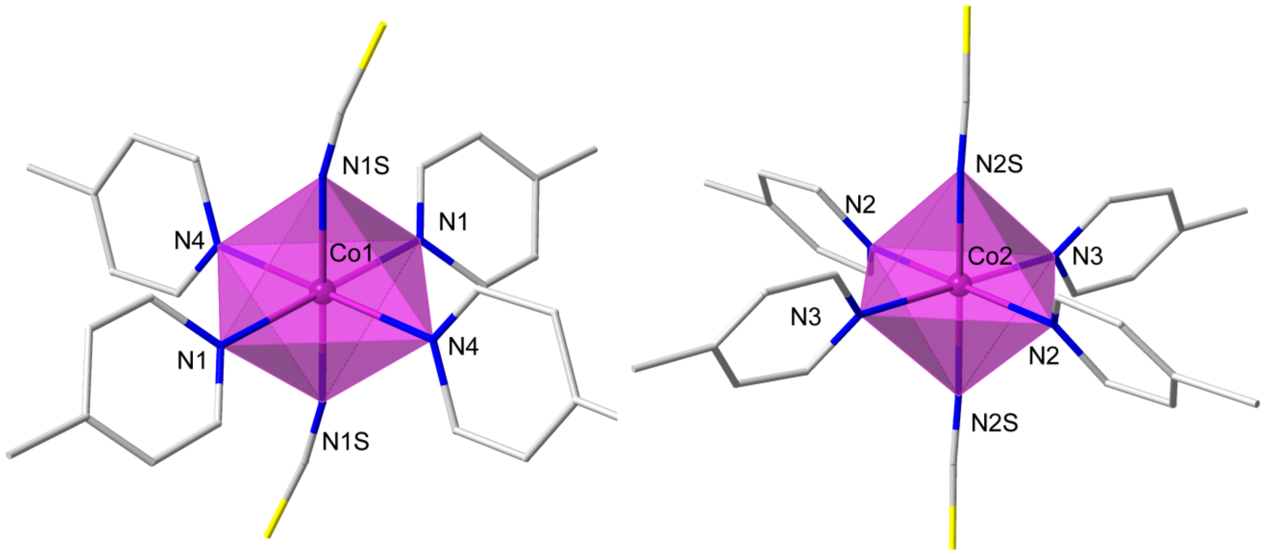

Fig. S5. View of the metal coordination polyhedron of **DCB@1** showing the tetragonally distorted geometry of the metal atoms. Cobalt and nitrogen atoms are represented with purple and blue colours, respectively.

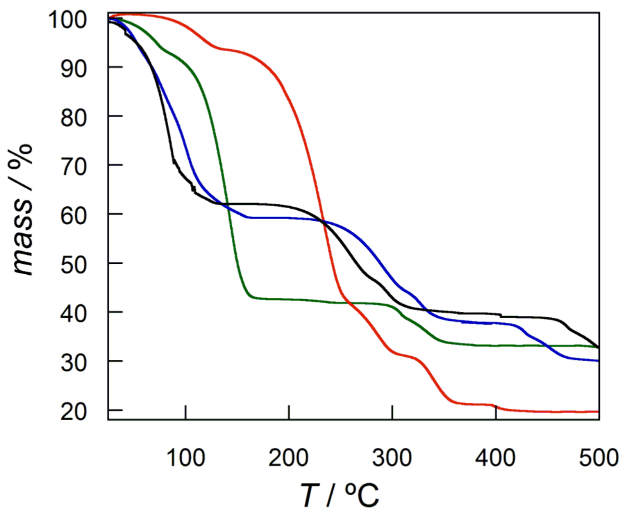

Fig. S6. TGA of **DCB@1** (green), **TAN@1** (red), **TOL@1** (blue) and **PYR@1** (black) under dry N_2 atmosphere.

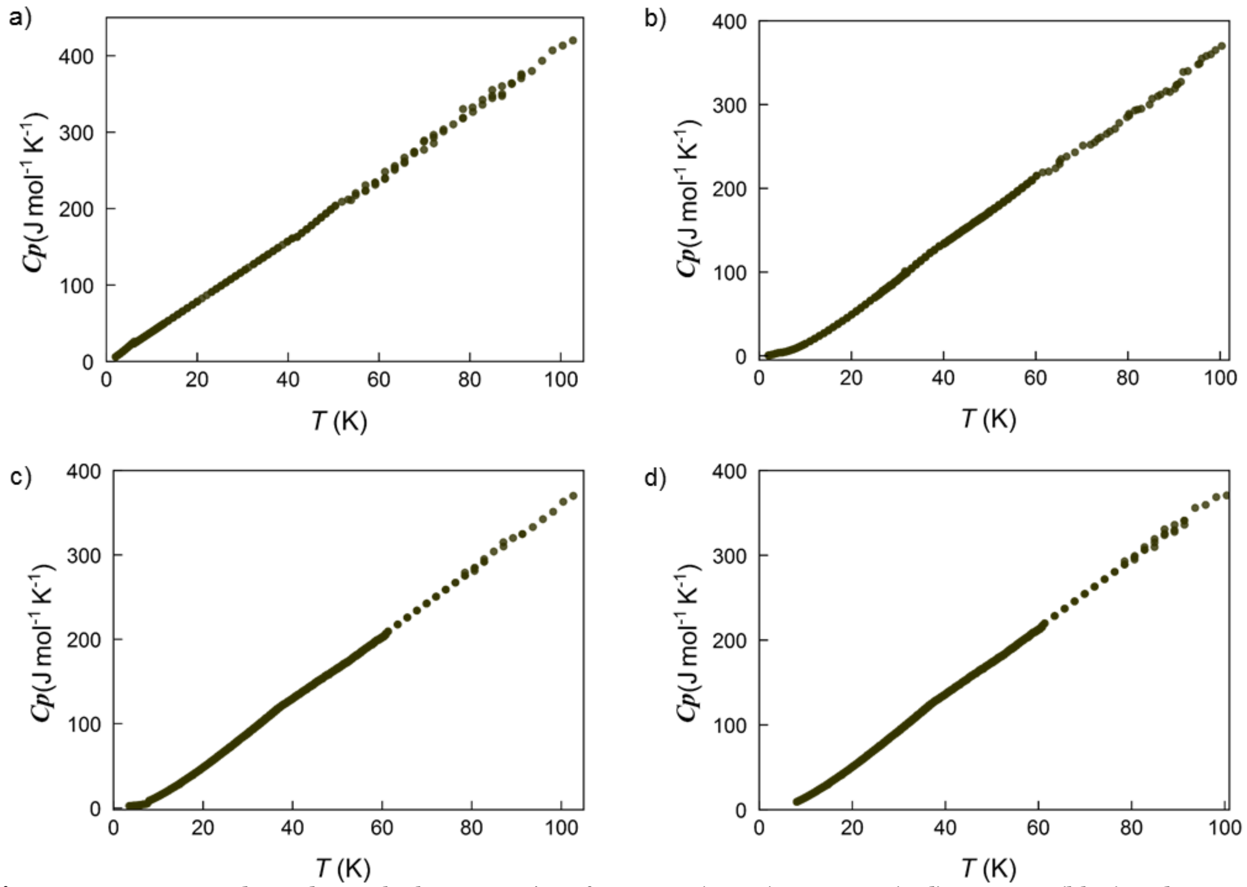

Fig. S7. Temperature dependence the heat capacity of **DCB@1** (green), **TAN@1** (red), **TOL@1** (blue) and **PYR@1** (black) in the range 2.0-100.0 K.

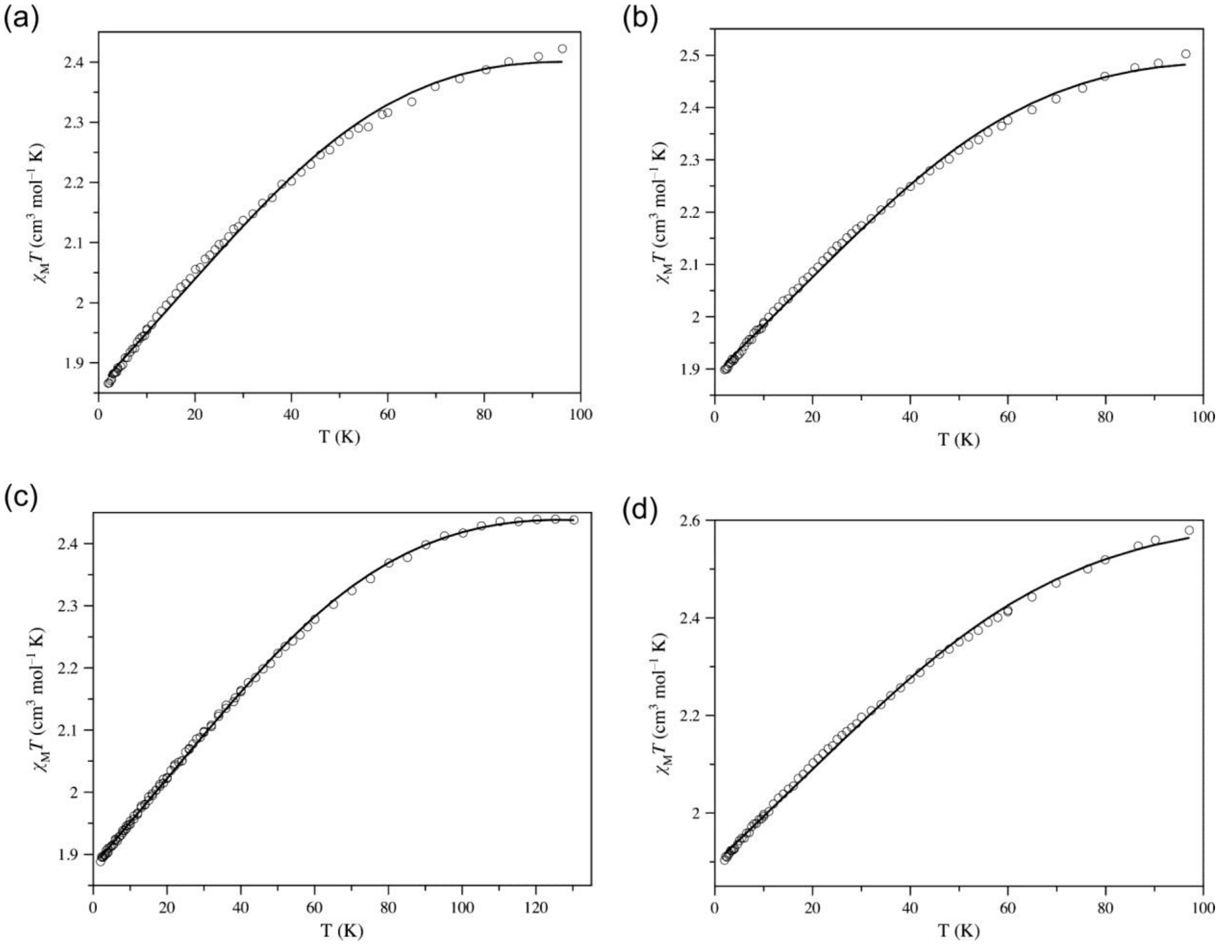

Fig. S8. Temperature dependence of $\chi_M T$ for **DCB@1** (a), **TAN@1** (b), **TOL@1** (c) and **PYR@1** (d) under an applied dc field of 0.1 (T < 30 K) and 1.0 kG ($T \geq 30$ K). The solid lines are the best-fit curves (see text).

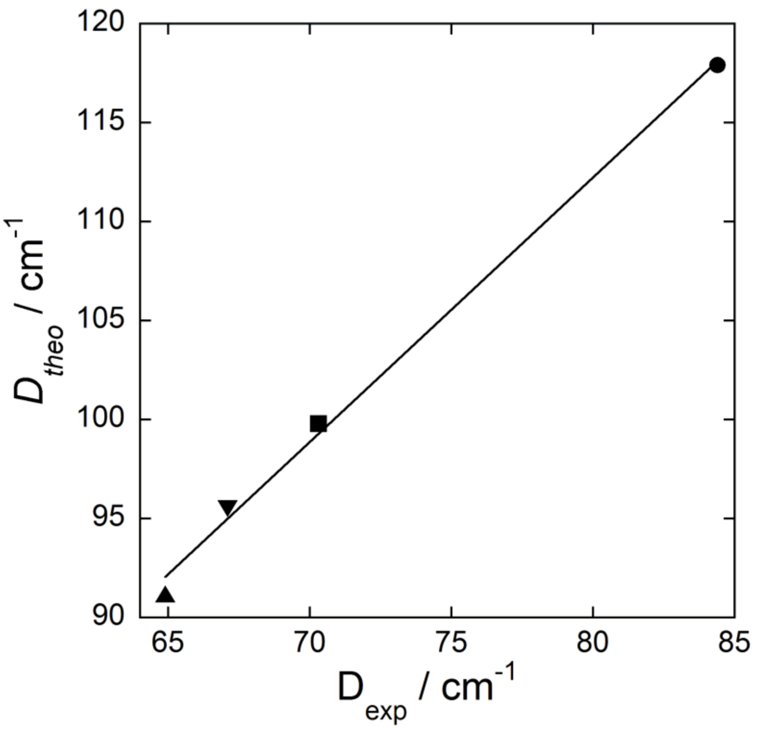

Fig. S9. Plot of the calculated *D* values (D_{theo}) *versus* the experimental ones (D_{exp}) for **DCB@1** (▲), **TAN@1** (▼), **TOL@1** (●) and **PYR@1** (■).

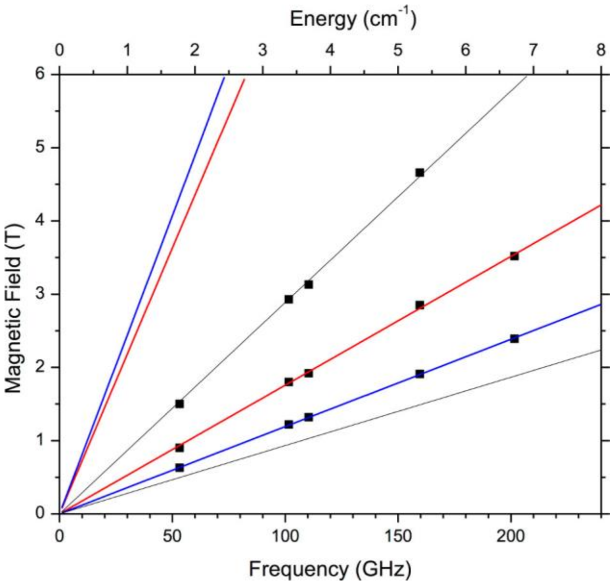

Fig. S10. Field vs. frequency map of the turning points in the HFEPR spectra of **TAN@1** at 4.5 K. The squares are experimental points while the lines were simulated using best-fitted spin Hamiltonian parameters: S = 3/2; $|E/D|$ = 0.125, g = [2.52, 2.56, 2.59]. Different colors mark particular turning points: red – magnetic field B_0 parallel to the x axis of the zfs tensor, blue – $B_0 \parallel y$, black – $B_0 \parallel z$. The lines with no experimental points on them represent transitions within the excited $|S, M_S\rangle = |3/2, \pm 3/2\rangle$ Kramers doublet that is not populated at low T.

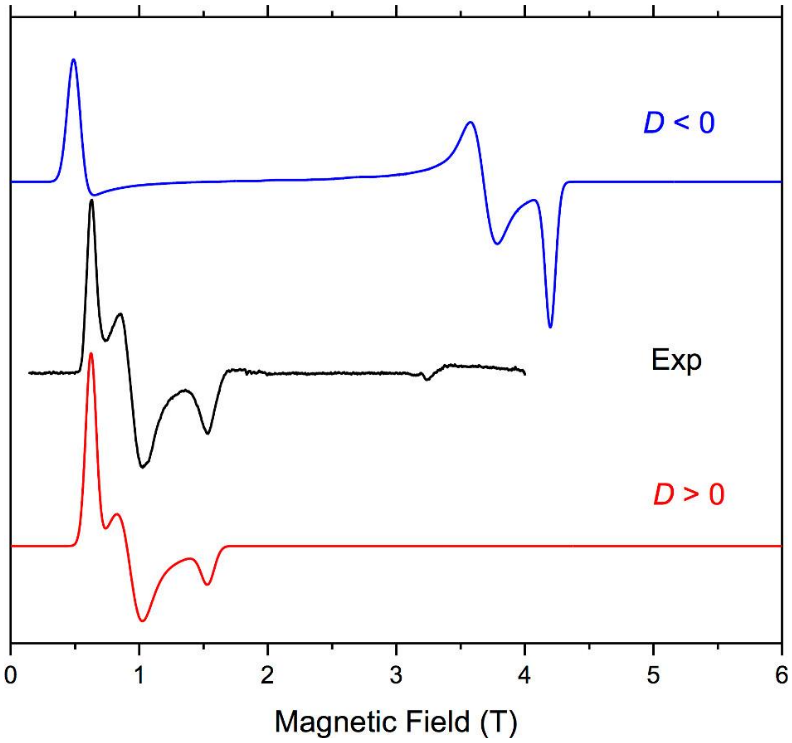

Fig. S11. EPR spectrum of **DCB@1** at 53.2 GHz and 4.5 K (black trace) accompanied by powder-pattern simulations (colored traces) using following spin Hamiltonian parameters: S = 3/2; $|E/D|$ = 0.13, g = [2.55, 2.55, 2.60].

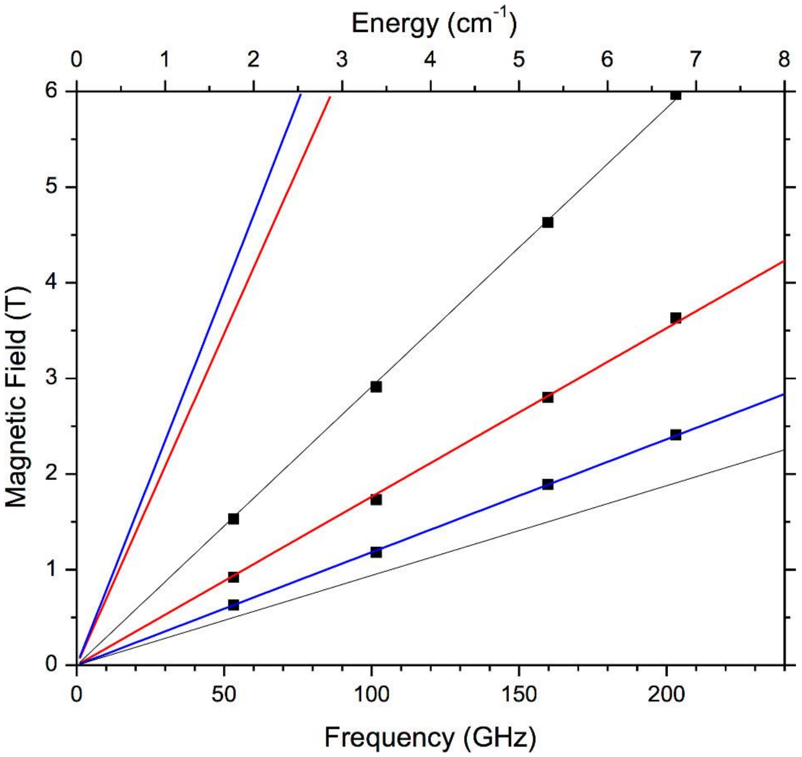

Fig. S12. Field vs. frequency map of the turning points in the HFEPR spectra of **DCB@1** at 4.5 K. The squares are experimental points while the lines were simulated using best-fitted spin Hamiltonian parameters: S = 3/2; $|E/D|$ = 0.13, g = [2.54, 2.56, 2.59]. Different colors mark particular turning points: red – $B_0 \parallel x$, blue – $B_0 \parallel y$, black – $B_0 \parallel z$.

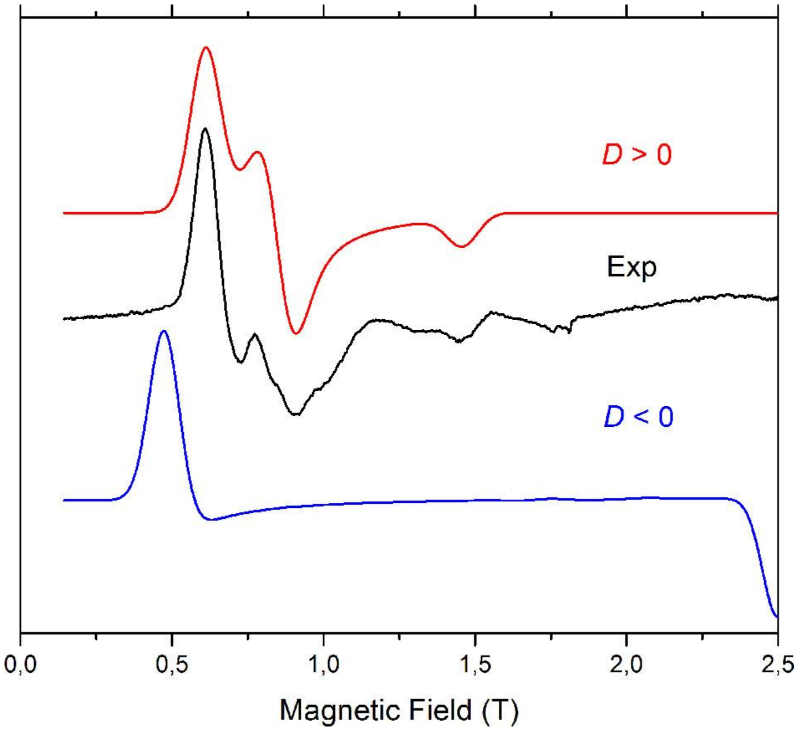

Fig. S13. EPR spectrum of **TOL@1** at 50.4 GHz and 4.5 K (black trace) accompanied by powder-pattern simulations (colored traces) using following spin Hamiltonian parameters: $S = 3/2$; $|E/D| \approx 0.11$, g = 2.55 (isotropic).

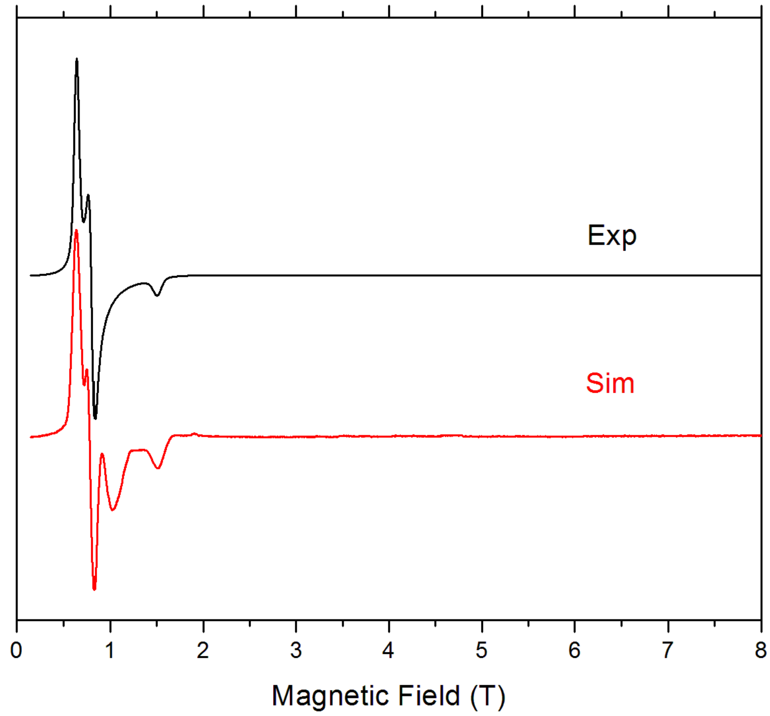

Fig. S14. EPR spectrum of **PYR@1** at 54 GHz and 4.5 K (black trace) accompanied by powder-pattern simulations (colored traces) using following spin Hamiltonian parameters: S = 3/2; D > 0, $|E/D|$ = 0.075, g = [2.7, 2.7, 2.6]. The peak at ~ 1.03 T is not simulated and assumed either due to an imperfect powder pattern, or a decomposition product caused by grinding. For this small rhombicity factor, the complex would be 'EPR-silent' in the 0 – 8 T field range if D < 0 hence no simulations are shown for such a case.

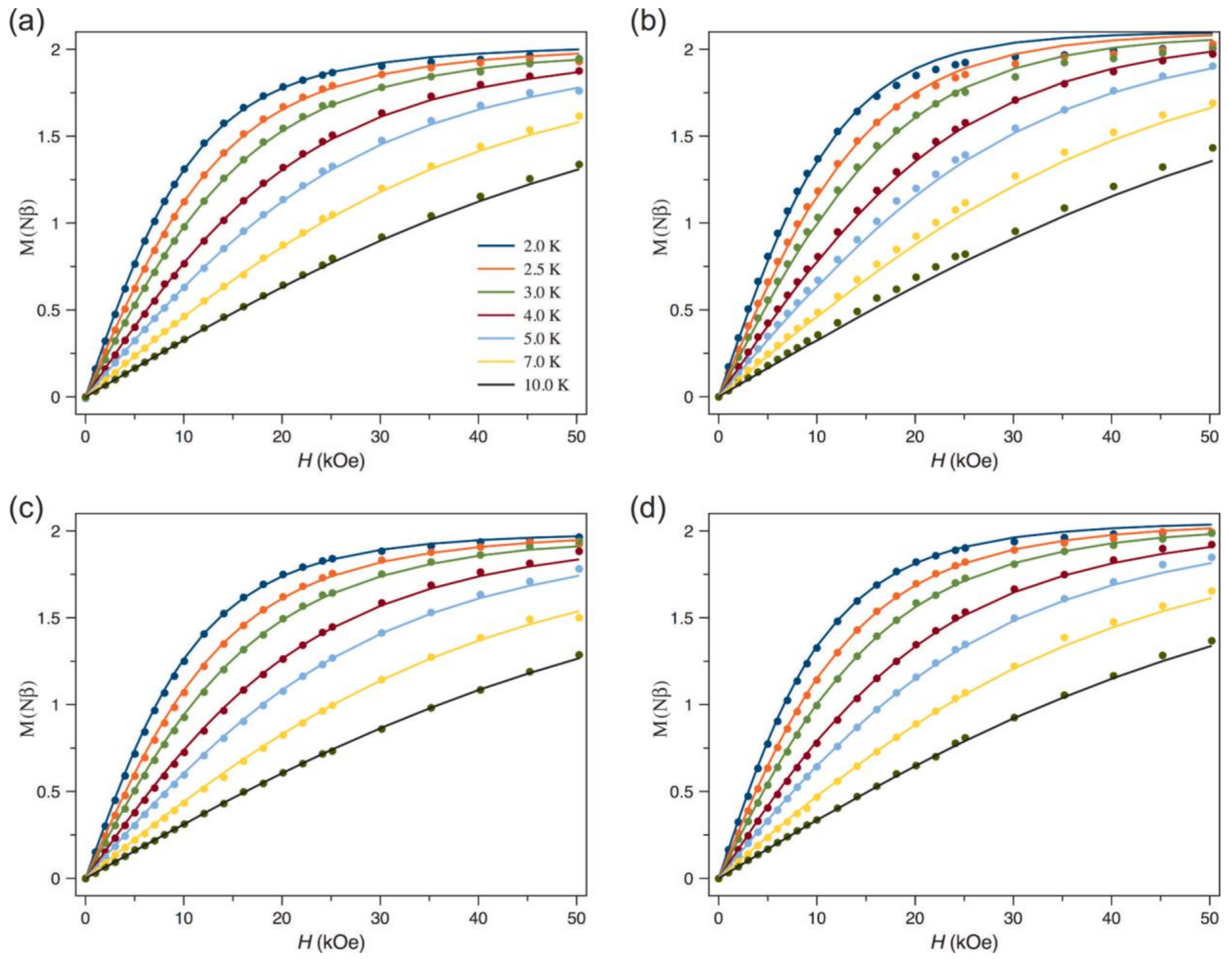

Fig. S15. H dependence of M of **DCB@1** (a), **TAN@1** (b), **TOL@1** (c) and **PYR@1** (d) in the temperature range of 2.0–10.0 K. The solid lines are the simulations with the parameters obtained from HFPER spectra for **DCB@1** and **TAN@1** and the best-fit curves for **TOL@1** and **PYR@1** (see text). An isolated ground state effective S = ½ Kramer doublet was considered in both cases.

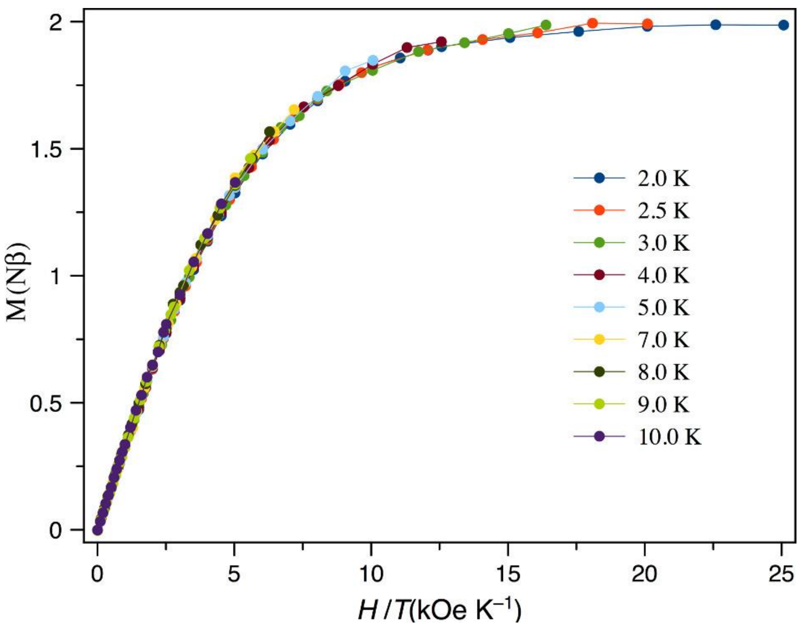

Fig. S16. Dependence of M with H/T of **PYR@1** in a temperature range of 2.0–10.0 K. The solid lines serve as an eye-guide.

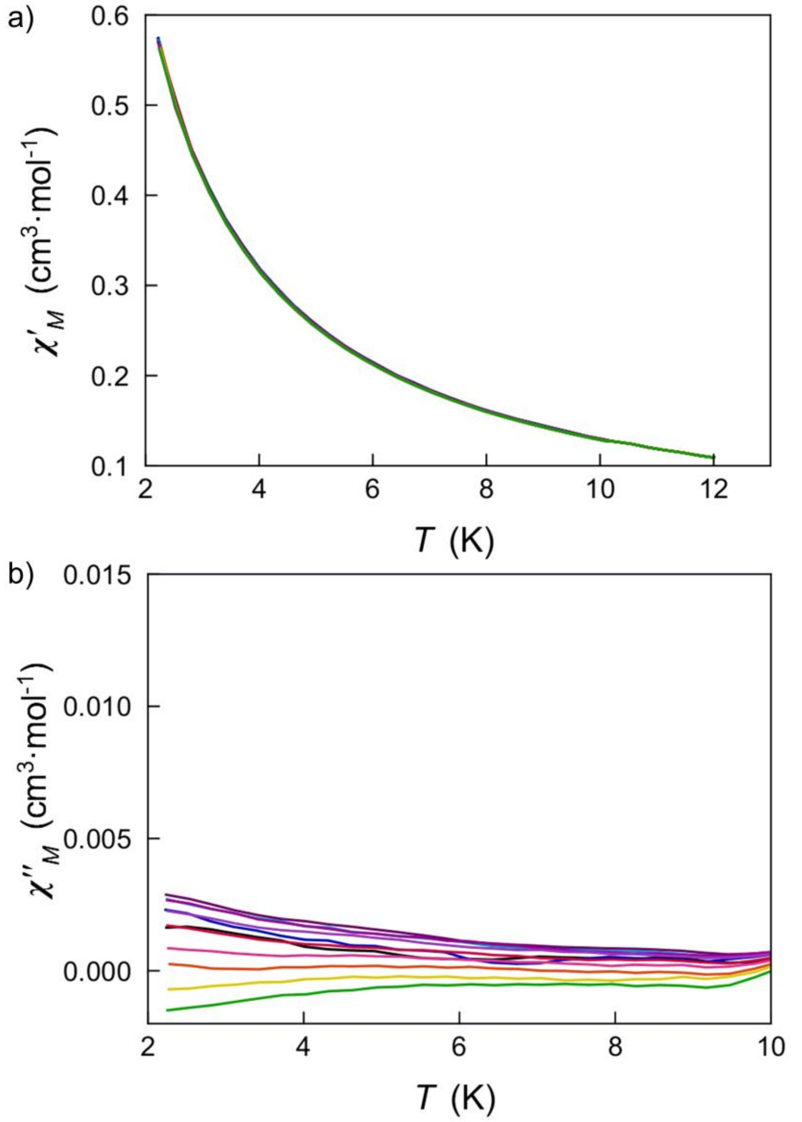

Fig. S17. Temperature dependence of χ_M' (a) and χ_M'' (b) of **DCB@1** in a 0 G applied static field and under 5.0 G oscillating field in the frequency range of 0.1–10 kHz.

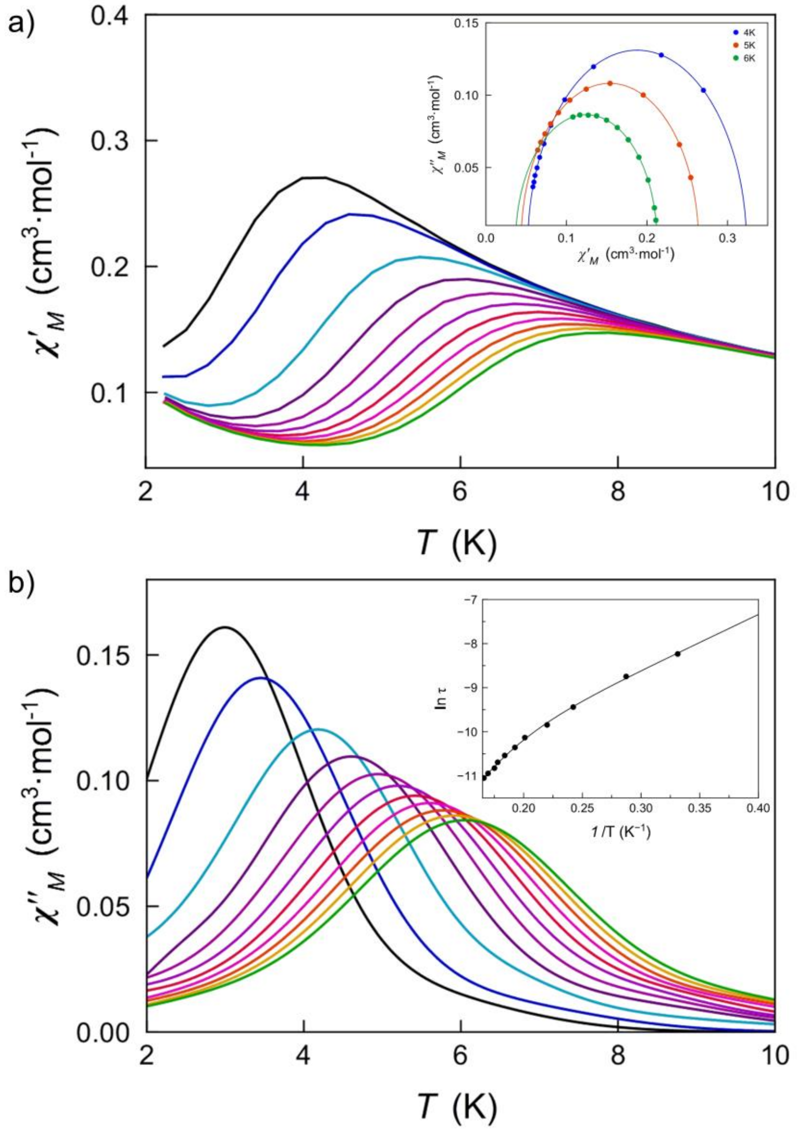

Fig. S18. Temperature dependence of χ_M‘ (a) and χ_M“ (b) of **DCB@1** in a 500 G applied static field and under 5.0 G oscillating field in the frequency range of 0.1–10 kHz. The insets show the Cole-Cole plots at 4.0, 5.0 and 6.0 K (a) and the Arrhenius plot (b) in the high temperature region. The solid lines are the best fit curves (see Table S2).

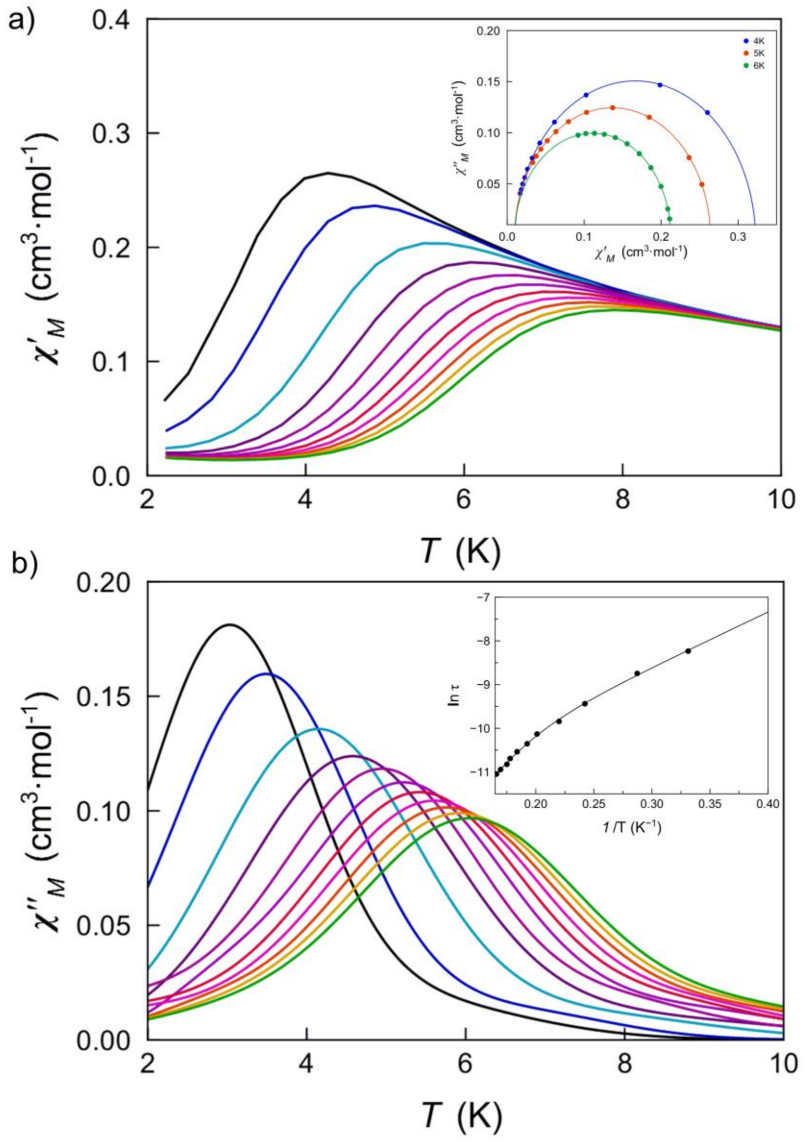

Fig. S19. Temperature dependence of χ_M' (a) and χ_M'' (b) of **DCB@1** in a 1.0 kG applied static field and under 5.0 G oscillating field in the frequency range of 0.1–10 kHz. The insets show the Cole-Cole plots at 4.0, 5.0 and 6.0 K (a) and the Arrhenius plot (b) in the high temperature region. The solid lines are the best fit curves (see Table S2).

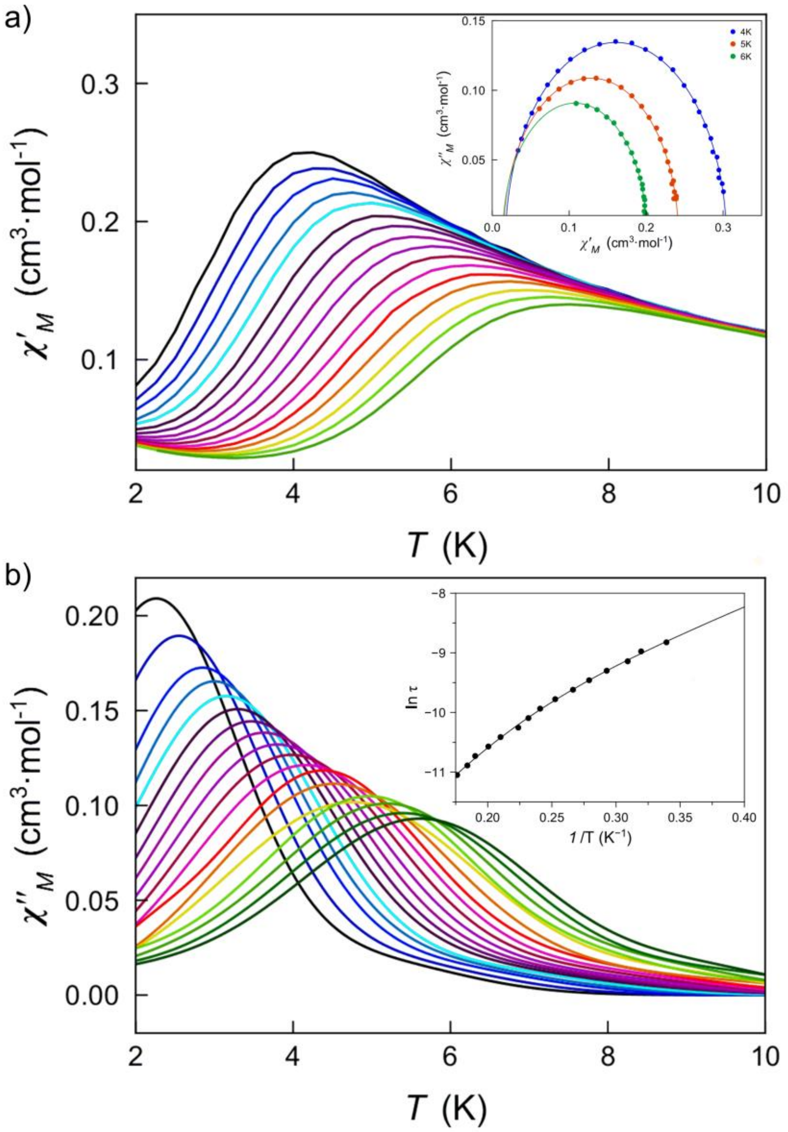

Fig. S20. Temperature dependence of χ_M‘ (a) and χ_M“ (b) of **TAN@1** in a 1.0 kG applied static field and under 5.0 G oscillating field in the frequency range of 0.1–10 kHz. The insets show the Cole-Cole plots at 4.0, 5.0 and 6.0 K (a) and the Arrhenius plot (b) in the high temperature region. The solid lines are the best fit curves (see Table S2).

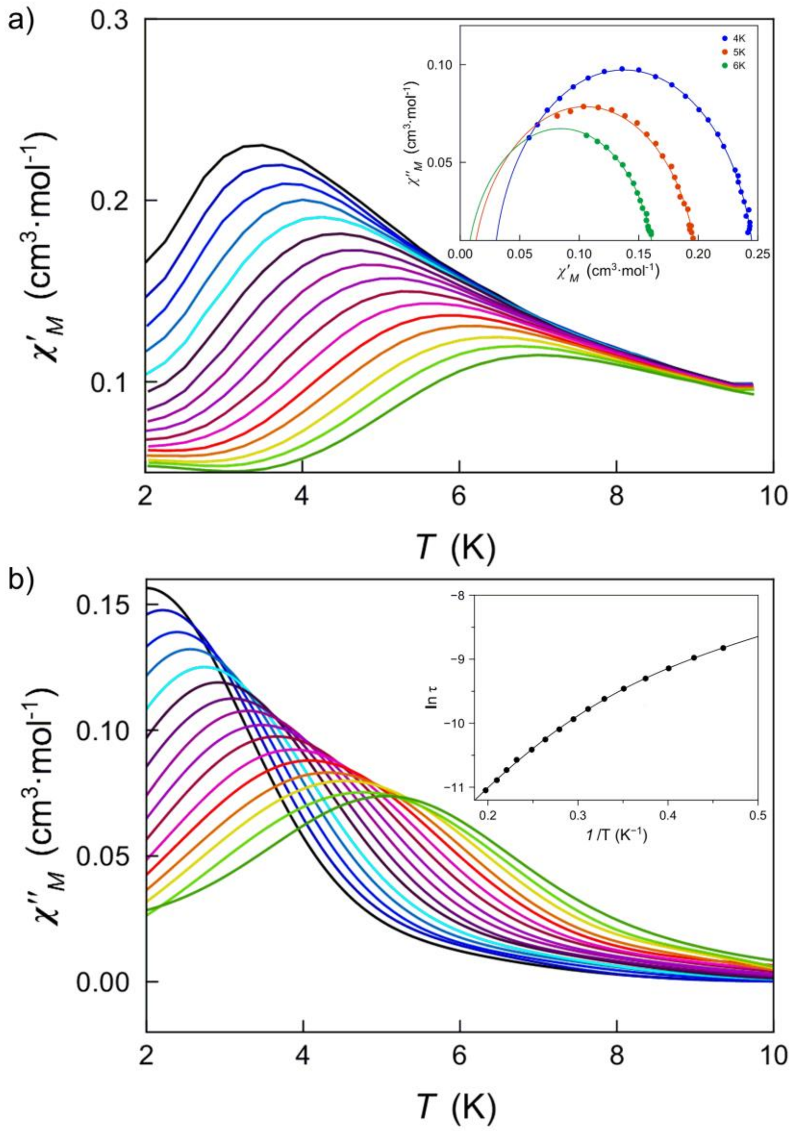

Fig. S21. Temperature dependence of χ_M' (a) and χ_M'' (b) of **TOL@1** in a 1.0 kG applied static field and under 5.0 G oscillating field in the frequency range of 0.1–10 kHz. The insets show the Cole-Cole plots at 4.0, 5.0 and 6.0 K (a) and the Arrhenius plot (b) in the high temperature region. The solid lines are the best fit curves (see Table S2).

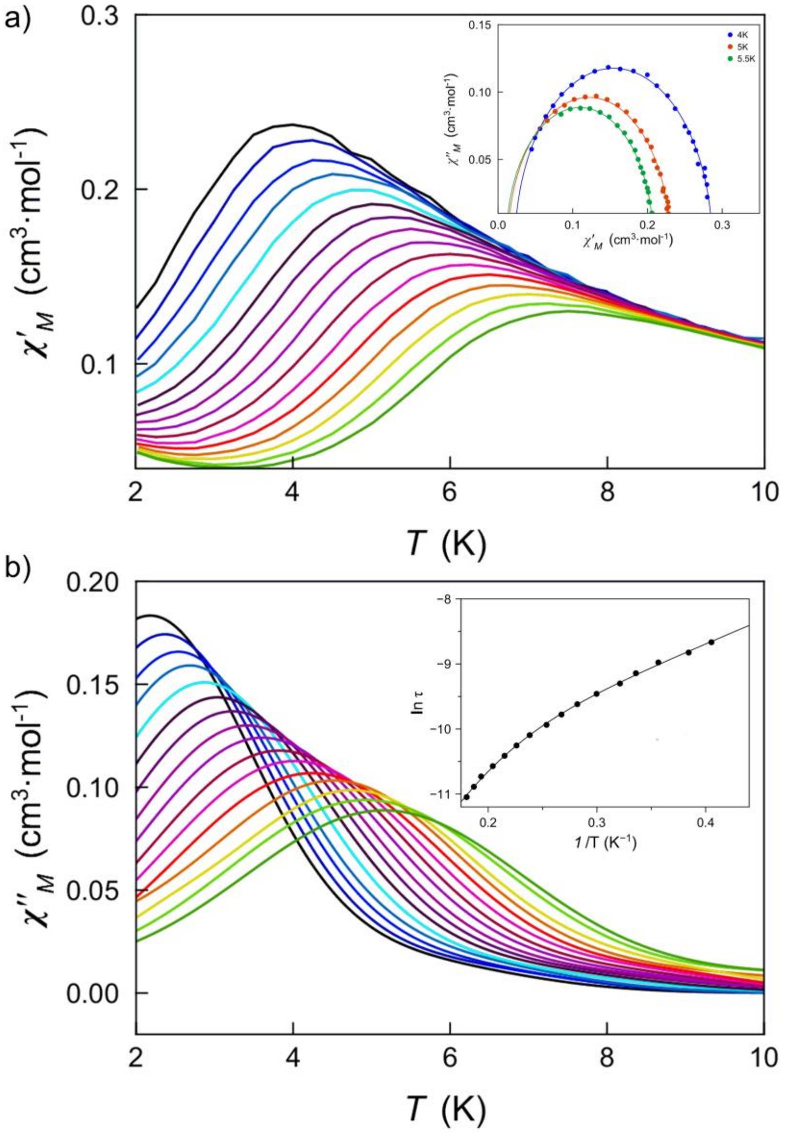

Fig. S22. Temperature dependence of χ_M' (a) and χ_M'' (b) of **PYR@1** in a 1.0 kG applied static field and under 5.0 G oscillating field in the frequency range of 0.1–10 kHz. The insets show the Cole-Cole plots at 4.0, 5.0 and 5.5 K (a) and the Arrhenius plot (b) in the high temperature region. The solid lines are the best fit curves (see Table S2).

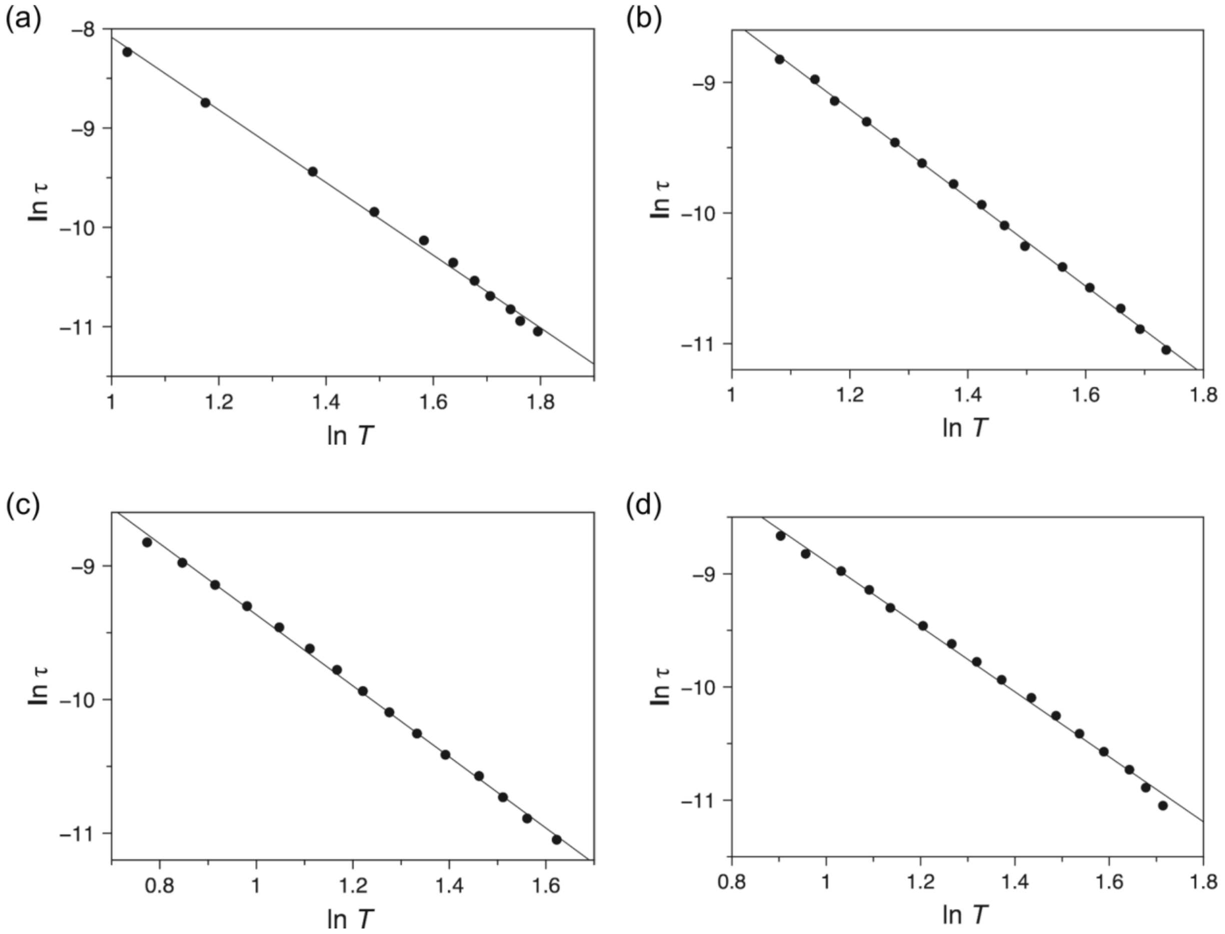

Fig. S23. The power-law probability distributions of relaxation times (τ) with the temperature (T) of **DCB@1** (a), **TAN@1** (b), **TOL@1** (c) and **PYR@1** (d) in a 1.0 kG applied static field. The solid lines are the best fit using the model $\tau^{-1} = AT^n$ (see text). The values of the exponent of the power-law (n) are shown in Tables S4.
